# Navigating the neuronal recycling bin: Another look at huntingtin in coordinating autophagy

**DOI:** 10.1080/27694127.2025.2472450

**Published:** 2025-06-02

**Authors:** Thomas J. Krzystek, Shermali Gunawardena

**Affiliations:** Department of Biological Sciences, The State University of New York at Buffalo, Buffalo, New York, USA

**Keywords:** Neurons, autophagy, huntingtin, huntington’s disease

## Abstract

Neurons, as post–mitotic and long–lived cells, rely heavily on autophagy to maintain cellular homoeostasis and ensure proper function. Huntingtin (HTT), a protein central to Huntington’s disease (HD), has emerged as a putative multifunctional regulator within the neuronal autophagy–lysosome pathway. This review explores normal HTT’s multifaceted role in neuronal autophagy, from its potential involvement in autophagy induction, its capacity to influence cargo recognition and autophagosome formation, and its contribution to autophagosome–lysosome fusion and transport. We also discuss the unique challenges that neurons face in maintaining proteostasis through autophagy, emphasising the need for specialised mechanisms like axonal transport of autophagosomes and distinct regulatory pathways. Furthermore, we highlight the spatial and temporal regulation of neuronal autophagy, particularly in the context of ageing and neuronal maturation, underscoring the importance of understanding HTT’s role in different neuronal states. By elucidating the intricate relationship between HTT and neuronal autophagy, this review aims to shed light on specific mechanisms of action in autophagy that can be disrupted in neurodegenerative diseases including HD.

## Introduction

Neurons are post–mitotic, long–lived cells that undergo extensive metabolic demands. Unlike mitotic cells, neurons lack the ability to mitigate the accumulation of damaged proteins and organelles through cell division. Instead, they heavily depend on degradative pathways such as the ubiquitin–proteasome system [UPS, 1], the endolysosomal pathway [2], and macroautophagy/autophagy [3] to maintain cellular homoeostasis for proper neuronal health and function. Of these pathways, autophagy is a tightly regulated and highly conserved catabolic pathway across eukaryotes responsible for degrading protein aggregates, damaged organelles, and invading pathogens through a coordinated sequence of autophagy induction, phagophore elongation/assembly, cargo recruitment, and autophagosome–lysosome fusion [4]. Autophagy is essential for neuronal survival and axonal homoeostasis [5], with autophagy loss leading to neurodegeneration [6,7]. While autophagy is thought to be constitutively active in neurons under basal conditions [8], an age–related downregulation of genes essential for autophagy has been observed in the human brain [9]. Consequently, neurons experience a diminished capacity for clearing cellular debris as individuals age [10,11], likely contributing to the pathogenesis of age–related neurodegenerative disorders such as amyotrophic lateral sclerosis [ALS, 12, 13], Parkinson’s disease [PD, 14, 15], Alzheimer’s disease [AD, 16, 17], and Huntington’s disease [HD, 18, 19]. Elucidating the currently unknown regulatory mechanisms of constitutive neuronal autophagy could unlock promising new therapeutic avenues aimed at restoring neuronal functions in these deadly diseases.

HD is a devastating neurodegenerative disorder hallmarked by loss of striatal neurons [20], progressive motor dysfunction, and cognitive decline [21,22]. HD manifests from an abnormal expansion of CAG repeats (>39) in the polyglutamine (polyQ) tract of the Huntingtin (HTT) gene [23,24], with the number of CAG repeats underlying the disease onset in patients [25]. Research into HD has identified a critical role for autophagy dysfunction in disease progression. Studies in post–mortem brains of HD patients [26,27], neurons differentiated from induced pluripotent stem cells (iPSCs) from HD patients [28], transgenic mice [29,30,31], mouse clonal striatal cells [32], rat pheochromocytoma (PC12) cells [32], and fly (Drosophila) HD models [33] revealed evidence of impairments in the autophagy–lysosome pathway. Given that toxic gain–of–function [34], loss–of–function [35,36], and aberrant polyQ–mediated events likely contribute differently to HD pathogenesis, there is significant uncertainty about how autophagy defects arise in HD [18]. This ambiguity is further complicated by mounting evidence that normal or wild type HTT itself might scaffold key machinery throughout different steps of the autophagy–lysosome pathway, including autophagy induction [33], cargo loading for selective autophagy [33,37], autophagosome formation [38], autophagosome movement [39], and the potential overlap with the endolysosomal system [29,40]. In this review, we will explore the multifunctional roles of normal HTT within the autophagy–lysosome pathway.

## The autophagy–lysosome pathway within neurons

Autophagy is a highly regulated cellular process that involves the degradation of damaged organelles and misfolded proteins through the formation of double–membrane vesicles called autophagosomes [4]. These autophagosomes fuse with lysosomes, where their contents are broken down and recycled, maintaining cellular proteostasis and promoting cell survival. Though key studies have laid the foundation for our understanding of the autophagy–lysosome pathway, there is still a great deal of ambiguity regarding autophagy in neurons. Here we will aim to define autophagy through the lens of a broad array of eukaryotic cells to highlight how neuronal autophagy likely faces unique challenges during autophagy induction, phagophore assembly, cargo recruitment, autophagosome fusion, transport, and maturation.

### Autophagy induction

The initiation of autophagy is thought to be regulated by the mammalian target of rapamycin 1 (mTOR), which inhibits the process under nutrient–rich conditions across most mammalian cells. The core regulatory complex for autophagy induction is comprised of unc–51–like autophagy activating kinase (ULK1), focal adhesion kinase family interacting protein of 200 kDa (FIP200), AuTophaGy–related 13 (ATG13), and ATG101. Under nutrient–rich conditions, mTOR phosphorylates ULK1 at Ser757 [41] and/or Ser638 [42], thereby suppressing ULK1ʹs catalytic activity and preventing autophagy [41]. Additionally, mTOR phosphorylates ATG13 [43] which further inhibits nutrient–dependent autophagy. Conversely, rapamycin–mediated mTOR inhibition causes decreased ULK1 and ATG13 phosphorylation [42,43], triggering autophagy and the activation of other components of the ULK1 complex. ULK1 activity seems to be regulated by competing mechanisms involving either mTOR or AMP–activated kinase (AMPK) depending on nutrient availability [41]. Indeed, AMPK can phosphorylate and activate ULK1 following mTOR inhibition [41].

However, this mechanism of action for autophagy induction might require further investigations in neurons. While mouse cortical neurons exhibited nutrient deprivation–induced autophagy in an mTOR–dependent manner [44,45], mouse brain tissue did not show elevated autophagy induction in response to starvation [8,46]. However, autophagy induction upon intermittent fasting suggests mechanisms might be more nuanced in brain tissue [46]. Moreover, rapamycin does not seem to consistently induce autophagy in cultured neurons: mouse cortical neurons exhibited short–term autophagy induction with one hour of rapamycin treatment [47] but failed to demonstrate autophagy induction with 48 hours of treatment [48]. Although mTOR–dependent autophagy might play a key role in neuronal events such as synaptic pruning [49] and synaptic transmission [50], mounting evidence suggests that alternative regulatory mechanisms likely exist via kinases capable of phosphorylating ULK1, such as AMPK [51], glycogen synthase kinase 3β [GSK3β, 52], and protein kinase Cα [PKCα, 53]. Moreover, neuronal autophagy seems to be capable of induction following activation of transcription factor EB (TFEB) in an mTOR/ULK1–independent manner [54]. This body of work underscores significant gaps in our understanding of how neuronal autophagy induction is achieved and highlights a need to explore alternative pathways involved in its regulation.

### Phagophore elongation/assembly

Once activated, the ULK1 complex interacts with a nucleation complex that includes Beclin–1, ATG14, and vacuolar protein sorting 34 (VPS34), a class III phosphatidylinositol 3–kinase [55,56,57], to generate phosphatidylinositol 3–phosphates (PI3Ps) essential for phagophore assembly [58]. Phagophores are a precursor to the autophagosome, and PI3Ps are also essential for recruiting proteins essential for autophagosome functions, such as WD repeat domain phosphoinositide–interacting proteins (WIPIs), Microtubule–associated protein 1A/1B–light chain 3 (LC3), and FYVE (Fab–1, YGL023, VPS27, and EEA1) domain–containing proteins [59,60]. Elongation of the phagophore is a complex process that relies on a variety of ATGs and a robust supply of lipids. Elongation involves two ubiquitin–like conjugation systems. In the first system, ATG7 (an E1–like enzyme) and ATG10 (an E2–like enzyme) conjugate ATG12 to ATG5 [61,62]. The ATG12–ATG5 conjugate, along with ATG16, acts as an E3–like enzyme to facilitate the conjugation of LC3–I (a mammalian ortholog of Atg8) to phosphatidylethanolamine (PE) to produce LC3–II [63]. This conjugation is critical for the expansion and curvature of the autophagosome membrane; however, it is relatively short lived as ATG5 and ATG12 can dissociate swiftly from the elongating phagophore following its biogenesis into an autophagosome [64]. The second conjugation system involves ATG7 and ATG3, which facilitate the processing and attachment of LC3 to PE, further aiding in membrane elongation and autophagosome maturation [65].

A vital element of autophagy is the supply of lipids for the growing autophagosome membrane. ATG9 is a protein with a transmembrane domain that plays a central role in this process by transporting lipids between organelles and the expanding autophagosome. The specialised structure at the donor membrane is referred to as the omegasome. The role of ATG9 is complemented by proteins like transmembrane protein 41B (TMEM41B), which localises to the endoplasmic reticulum (ER) and are involved in lipid transfer to the phagophore [66]. Interestingly, ATG9 has been observed to localise across a wide range of membranous structures such as ER [67], mitochondria [68], endosomes [69], golgi [70], and synaptic vesicles [71]. Unsurprisingly, a wide variety of membrane sources have been implicated in autophagy: plasma membrane [72], mitochondria [73], ER [74,75], Golgi [76], and endosomes [77]. Since ATG9–mediated lipid transfer is essential for the elongation and closure of the autophagosome, there is a need to understand how a neuron regulates the distribution of ATG9 throughout its extensive axonal network.

ER is a complex organelle canonically classified into two categories based on the absence or presence of membrane–associated ribosomes. While rough ER is ribosome–studded and involved in translation and protein–folding, smooth ER is void of ribosomes and plays more homoeostatic roles such as calcium buffering and lipid synthesis. Although rough ER has not been observed within axonal projections and is primarily localised to the somatodendritic compartment of neurons, smooth ER is thought to pervade axonal projections as narrow, continuous, and inter–connected tubules [78,79,80]. While axonal ER functions are still being explored in the context of the secretory pathway [81,82], synaptic transmission [83], and microtubule–dependent transport [84,85,86], work in mouse hippocampal neurons revealed that autophagosomes in distal neurites can form locally and colocalize with ER [64]. This supports the proposal that neurons likely utilise axonal ER as lipid donors for local autophagosome biogenesis throughout their long and complex processes; however, it remains uncertain if this is a compensatory mechanism due to the metabolic demands of neurite outgrowth following dissociation. Indeed, components of the endolysosomal system have been suggested to fuse with the plasma membrane of distal neurites to provide new lipids for extending neurite processes [87,88]. Furthermore, it remains uncertain how these protein complexes involved in phagophore elongation and assembly are moved within axons. Though loss of ATG5 in cultured mouse neurons disrupted the axonal movement of ER (labelled by the ER–localisation KDEL motif) and LC3–labelled autophagosomes [50], it is unknown how ATG5 and other members of this phagophore assembly complex are moved/distributed within axons. As we elucidate these mechanisms underlying neuronal autophagy, it is critical that distinctions between developing neurons with growth cones and matured neurons with established synapses are assessed since these states represent distinct environments with specific functional needs.

### Cargo recruitment

Through the formation of double–membraned vesicles called autophagosomes, autophagy engulfs and delivers these components to lysosomes for degradation. Depending on the cargo being degraded, autophagy can be classified as either “non–selective” or “selective”. While non–selective autophagy typically involves starvation–induced bulk degradation of cytosolic components to assure adequate nutrients for cellular functions, selective autophagy involves the recruitment of specific cargo (ie: toxic protein aggregates and damaged organelles) recognised by cargo receptors [89] that are characterised by their ability to associate with autophagosomes via LC3–interacting region (LIR) motifs [90]. Though different cargo receptors have been studied in the context of different subtypes of selective autophagy (ie: mitophagy versus aggrephagy), p62/sequestosome 1 [SQSTM1, 91, 92], neighbour of BRCA1 [NBR1, 93], Tax1 binding protein 1 [TAX1BP1, 94], nuclear dot protein 52 [NDP52, 95], and optineurin [OPTN, 39] are among the more extensively studied cargo receptors for selective autophagy. In the context of neurons, work over the last decade has identified that several proteins linked to neurodegenerative diseases may associate with cargo receptors and/or LC3 [96], highlighting putative mechanisms for autophagy defects that might be neuron specific. Indeed, repeat hexanucleotide expansions in C9orf72 are the most common genetic cause of ALS [97] and C9orf72 has been observed to associate with p62 to mediate the targeting of stress granules in mouse motor neurons for p62–dependent autophagy [98]. Though it is unclear how C9orf72 recruits p62 and other autophagy machinery to help motor neurons eliminate stress granules, this work raises the question of whether this is a neuron–specific pathway to mitigate compounding stress due to a neuron’s long–lived, post–mitotic life.

Cargo receptors serve as a linker between ubiquitinated cargo, damaged organelles, and/or aggregated proteins and autophagosome–associated LC3–II, ensuring the specific incorporation of target molecules into the autophagosome. The selective degradation of ubiquitinated protein aggregates is known as aggrephagy, and p62 is the most characterised cargo receptor that is involved in recognising ubiquitin chains attached to protein aggregates [91], though NBR1 has also been shown to be involved [93]. In contrast, selective degradation of organelles, such as mitochondria, has been long thought to involve specific machinery. OPTN [39] and NDP52 [99] were shown to be critical for the selective degradation of mitochondria through a process known as mitophagy, which are recruited by the ubiquitin kinase PTEN–induced kinase 1 (PINK1) to subsequently recruit ULK1 and downstream autophagy machinery such as LC3 [99]. Though this process wasn’t thought to involve p62, there is mounting evidence that p62 may be involved in PINK1/Parkin–dependent mitophagy through a mechanism that involves NF–kB essential modulator (NEMO) which is distinct from NDP52 and OPTN [100,101]. This overlapping function of p62 highlights that these cargo receptors might not be reliable markers to distinguish between different types of autophagy and likely require tandem investigations to validate the pathways involved. Therefore, there remains a need to investigate overlapping and distinct regulatory mechanisms between the differential involvement of cargo receptors such as p62 and OPTN during selective autophagy.

### Autophagosome fusion, transport, and maturation

Once an autophagosome is fully formed, it can mature by fusing endolysosomes, including late endosomes and lysosomes [102]. This fusion process is facilitated by complexes containing soluble N–ethylmaleimide–sensitive factor attachment receptors [SNAREs, 103], particularly syntaxin 17 [STX17; 104] and syntaxin 7 [STX7; 105]. In neurons, autophagosomes forming in distal neurite processes have been shown to require STX17–mediated fusion with RAB7 GTPase–containing endolysosomes, forming amphisomes, prior to retrogradely moving towards cell bodies [106]. Retrograde movement towards the soma has been shown to be critical for the maturation of amphisomes/autophagosomes in mouse hippocampal neurons [107]. However, there is conflicting evidence regarding its importance for fusion with lysosomes for degradation in mouse cortical neurons. [108,109], provided evidence that degradative lysosomes are enriched near the soma. In contrast, [110], reported that degradative endolysosomes can also be delivered to distal regions of neurites to maintain local degradation capacity. Contributing to the uncertainty of whether the soma is a primary site for lysosomal degradation in neurons is the use of markers for these organelle structures that might not be as discrete as originally thought. LC3, the primary marker used to monitor the dynamics of autophagosomes [111], is present across multiple phases of the autophagy pathway beginning with phagophore assembly and ending with lysosomal fusion. Likewise, lysosome–associated membrane protein 1 (LAMP1), the predominant marker used to monitor lysosome dynamics, was shown in mouse cortical neurons to label a significant proportion of organelles that do not contain the hydrolytic enzymes and proteases necessary for degradation [108]. Therefore, while we need to be cautious about concluding the identity of organelles using these markers, there is a need to identify additional components of these autophagic and lysosomal structures to distinguish the specific compartments being investigated.

Late endosomes are predominantly characterised by the presence of RAB7–GTPase responsible for the coordination of late endosomal functions [112,113]. Although fusion of RAB7–containing endolysosomes with LC3–containing autophagosomes have been shown to be critical for retrograde movement and maturation, RAB7 can coordinate both the anterograde and retrograde movement of late endosomes/endolysosomes. During retrograde axonal movement, GTP–bound RAB7 can interact directly with RAB7–interacting lysosomal protein (RILP) at its effector–interacting switch region [114,115]. The RILP–RAB7 association has been shown to prevent RAB7 from hydrolysing GTP, thereby keeping it in the active state that is vesicle bound [115]. RILP localises to RAB7 by binding the VPS41 subunit of the homotypic fusion and protein sorting (HOPS) complex [116]. RILP was shown to aid the recruitment of dynein/dynactin to RAB7–containing membranes through direct interaction with the p150glued C–terminus [117]. Moreover, oxysterol–binding protein–related protein 1 L (ORP1L) simultaneously binds RAB7 and RILP to stabilise their interactions with dynein/dynactin [117,118,119]. Alternatively, RAB7 mediates anterograde movement through the recruitment of the FYVE and coiled–coil domain–containing protein 1 [FYCO1/RUFY5; 120]. FYCO1 serves as a coincidence detector that can interact with both RAB7, PI3Ps, and LC3 [86,120], highlighting a potential mechanism in which RAB7–containing endolysosomes recruit LC3–containing autophagosomes. Additionally, FYCO1 has been shown to aid the recruitment of kinesin–1 to RAB7–containing membranes through direct interaction with kinesin–1 [120]. Although FYCO1–dependent anterogradely moving RAB7–containing endolysosomes were observed in projections of PC12 cells [86], the biological role of an anterogradely moving RAB7–containing vesicle within a neuron remains unclear. Perhaps these are endolysosomes contributing to the local degradation capacity in distal neurites [110], but further work will be needed to test these predictions in the context of a neuron.

## Normal, wild type huntingtin (HTT) as a multifunctional scaffold during autophagy

HTT is a very large 350 kDa protein that is ubiquitously expressed but enriched in the brain [121,122]. Though researchers identified HTT over thirty years ago to be the genetic cause of HD [123], the normal function of HTT within neurons remain elusive despite being essential for development with loss of HTT causing early embryonic lethality [124,125]. One proposed function of HTT is as a multifunctional scaffolding protein at membranes based on key components of its structure as well as its numerous interacting partners. Indeed, HTT has several major HEAT (Huntingtin, Elongator factor3, PR65/A regulatory subunit of PP2A, and Tor1) repeat domains, giving a solenoid–like shape [126], believed to enhance protein–protein interactions and scaffolding functions [127,128]. The sweeping presence of these HEAT repeat domains across the length of HTT supports scaffolding functions near HTT’s N– and C–terminal domains [129]. Moreover, the N–terminus of HTT exhibits a proline–rich region that enhances structural flexibility and aids protein–protein interactions [130]. Further evidence for a putative scaffolding role is the fact that HTT interacts with more than 350 partners isolated through yeast–two hybrid [Y2H; 131, 132] and mass spectrometry (MS) proteomics [133,134,135]. However, the biological relevance of this myriad of interactions remains unknown likely due to HTT’s complex cellular localisation pattern across membrane–bound organelles.

Biochemical analysis showed HTT to be at membranes [136], and HTT can associate with membrane phospholipids [137,138,139], likely by an amphipathic alpha helical membrane–binding domain in the first 17–18 amino acids of HTT’s N–terminus [139,140,141,142]. Membrane–bound HTT can function in a variety of cellular processes including neural adhesion [143], endocytosis [144], vesicle fusion [146], and transport [147,148]. Indeed, HTT localises to a variety of membranous subcellular compartments such as synaptic [136] and RAB–containing [40,149,150,151] vesicles, plasma membranes [139], ER, Golgi, endosomes [140,152], and autophagosomes [39]. Moreover, deletion of the N–terminal alpha helical structure in HTT resulted in decreased HTT localisation across ER, golgi, and vesicle membranes, increased nuclear accumulation, and induced cytotoxicity [140,153], highlighting the importance of these first 17–18 amino acids for HTT’s subcellular localisation across membranes.

**Figure 1.**
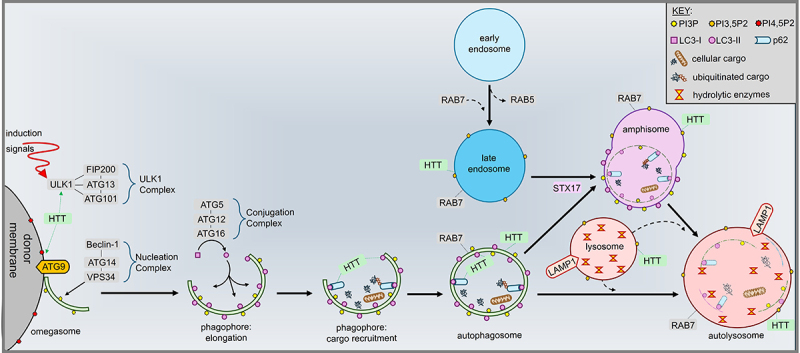
Schematic representation of HTT’s multifunctionality in the autophagy–lysosome pathway. HTT interacts with ULK1, aiding its recruitment to donor membranes containing PI4,5P2 and ATG9, leading to phagophore nucleation, and LC3–I conjugation to LC3–II. HTT can interact with p62 for cargo recruitment of cellular debris, damaged organelles, and ubiquitinated proteins. Subsequently, HTT associates with autophagosomes and late endosomes, likely acting in concert with RAB7 for microtubule–dependent transport to lysosomes following STX17–mediated fusion. Finally, HTT is implicated in lysosome fusion with both autophagosomes and amphisomes for the degradation of cellular cargo.

HTT may preferentially associate with membranes depending on the local populations of phosphoinositide (PI) phospholipids, with a strong bias for phosphatidylinositol 3,5–bisphosphate (PI3,5P2) and phosphatidylinositol 4,5–bisphosphate (PI4,5P2) observed in vitro [139]. Endolysosomes are heavily decorated with PI3,5P2 [154,155], which are produced from PI3Ps by PIKfyve in mammals [156]. Loss of PIKfyve, and subsequently PI3,5P2 was observed to be embryonically lethal in knockout mice [157]. Although PIKfyve and PI3,5P2 are critical for the distribution and motility of endolysosomes in human cells and mouse fibroblasts [158], their regulation, localisation, and downstream effect on the autophagy–lysosome pathway in neurons remain obscure. Since reliable live–cell reporters for PI3,5P2 have only recently been discovered [159], we are now better equipped with tools to help clarify how PI3,5P2 helps coordinate neuronal autophagy and how PI3,5P2–containing organelles are regulated within the complex architecture of a neuron. Although PI4,5P2 are heavily produced and localised at plasma membranes [160], they have also been identified at other organelles such as autophagosomes [161], perhaps acquired through fusion with endolysosomes following endocytosis from the plasma membrane [162,163]. While PI4,5P2 has been proposed to play a role in upstream autophagy induction – before PI3Ps – by regulating the recruitment of ATG14 [164], PI4,5P2 has been well studied in the context of autophagosome fusion with lysosomes [165]. Indeed, disrupted generation of phosphatidylinositol 4–phosphate (PI4P) resulted in defective lysosome–autophagosome fusion via RAB7 inactivation in human HEK293 cells [166]. Although HTT has been shown to form a complex with RAB7 on endolysosomes in mature fly axons Figure 1; [40, 150], the upstream events that dictate HTT’s localisation across these membranous compartments remain unclear. With recent advances in our understanding of PI3,5P2 and PI4,5P2 in the autophagy–lysosome pathway, it is also possible to predict that these unique lipids can help recruit HTT to these organelles (Figure 1).

HTT’s localisation and membrane trafficking can also be regulated by post–translational modifications, including palmitoylation at Cys214 [167]. Interactions with the palmitoyl–acyl transferases (PATs) HIP14 (ZDHHC17) and HIP14L (ZDHHC13) facilitate HTT palmitoylation [167]. Furthermore, HTT may also play a role in regulating HIP14/HIP14L–mediated palmitoylation of other proteins since reduced HIP14 PAT activity was observed in mice exhibiting an antisense oligonucleotide–mediated HTT knockdown [169]. Further evidence in Y2H supports this, revealing a strong overlap between HTT and HIP14 interactors [170]. While palmitoylation helps regulate the localisation of both normal and pathogenic HTT [167], as well as the localisation of pre–synaptic vesicle proteins such as SNAP25 and GluR1, [170, 169, 171, 172], it remains to be tested whether HTT–mediated HIP14 PAT activity plays a role in the autophagy–lysosome pathway. Be that as it may, HIP14L (ZDHHC13) was observed in HeLa cells to palmitoylate ULK1 and siRNA–mediated reduction of ZDHHC13 showed disrupted autophagic flux and decreased abundance of LC3–containing cargo [173]. Therefore, it is possible that HTT palmitoylation and/or HTT–mediated HIP14/HIP14L PAT activity plays a key role in regulating the autophagy–lysosome pathway. Although overexpression of HIP14 in mice expressing pathogenic HTT has shown therapeutic promise [167], it remains to be tested whether this rescue involves alleviation of mutant HTT (mHTT)–mediated autophagy defects.

Over the last decade, conflicting evidence hints at a multifaceted role for HTT in the autophagy pathway. Work in fly and human (HEK293) cells revealed that HTT interacts with ULK1, implicating it in the upstream induction of autophagy Figure 1; [174, 33]. Furthermore, HTT may be involved in cargo recruitment through observed interactions with p62 [33,174] and LC3 [37] to facilitate the coordination of materials to the elongating phagophore (Figure 1). However, the subcellular localisation of HTT–ULK1 and HTT–p62 associations were not examined in neurons. In fly neurons with functional synapses, HTT was not observed to co–localise with ATG5 in axons [40]. This implicates that HTT’s role in axons might be distinct from its role with ULK1 in autophagy induction and with p62 in selective autophagy – perhaps at axon terminals and at the soma. Additionally, HTT interactions with HAP1 and the molecular motor proteins kinesin–1 and dynein/dynactin [130,148] supports a role in trafficking of autophagosomes and endolysosomes Figure 1; [39], likely to ensure their proper distribution within growing neurons. However, whether this is the case in a mature neuron is unknown. Furthermore, expression of excess HTT in mice revealed increased endolysosomal activity [29], highlighting HTT as supporting lysosomal clearance pathways. Moreover, structural properties of the HTT protein shares considerable similarities with three essential autophagy–related proteins: Atg23, Vac8, and Atg11 [175]. Additionally, a caspase–3–cleaved fragment of HTT was found to be similar to an autophagosome–targeting sequence domain of ATG14L shown to regulate autophagy by sensing and promoting membrane curvature [18]. Collectively, these reports implicate HTT as a key player in neuronal autophagy, orchestrating various stages of this essential cellular process (Figure 1).

## Challenges for autophagy in neurons and HTT’s physiological role

Being post–mitotic, neurons face unique challenges in maintaining cellular homoeostasis through autophagy. The task of degrading and recycling damaged proteins and organelles is further complicated by the extensive length of neuronal axons, which can extend up to 1 mm in humans. Further, the maintenance of cellular homoeostasis during neuronal growth where components need to be degraded quickly in contrast to established neurons can add to the complexity. Unlike mitotic cells, neurons likely adapted autophagic machinery and regulatory pathways to overcome these challenges. Overall, the neuron must conquer four major hurdles in the context of autophagy. These are (1) axonal transport of autophagosomes, (2) the distinct regulatory mechanisms of neuronal autophagy induction, (3) the spatial production of autophagosomes within a neuron, and (4) the temporal regulation of autophagy in an ageing neuron.

### Axonal transport of autophagosomes

Although autophagosomes are proposed to form distally in axons [72,176], degradative lysosomes capable of hydrolysing proteins and damaged organelles are reported to be enriched near the soma [108,109]. Even though evidence in mouse cortical neurons suggests that degradative lysosomes anterogradely traffic to distal axons to maintain local degradation capacity [110], disrupting the retrograde movement of autophagosomes and endolysosomes resulted in the aberrant accumulation of these cargo in axons [106]. This suggests that the local degradative capacity may not be sufficient for clearance of autophagy cargos and that retrograde movement to the soma is necessary for other events in the pathway such as recycling via Golgi [178]. It is well characterised that autophagosomes acquire machinery for retrograde movement via fusion with endolysosomes by STX17 [106]. HTT has been implicated in supporting the long–distance trafficking of various cargoes, including synaptic vesicles [148], endosomes [150,151], and autophagosomes [39]. Through associations with Huntingtin–associated protein 1 (HAP1) and the dynein/dynactin complex [179,180], HTT helps coordinate the movement of autophagosomes generated at the distal ends of axons towards the cell body for fusion with lysosomes [39]. HTT is also involved in forming a complex with RAB7 on retrogradely moving endolysosomes that contained STX17 [40], Figure 1. Despite this knowledge, the precise mechanisms coordinating the recruitment of autophagosome–endolysosome fusion machinery in distal axons remain unclear. Evidence suggests that autophagosomes and amphisomes may use sequential adaptors, such as c–Jun N–terminal kinase–interacting protein 1 (JIP1), c–Jun N–terminal kinase–interacting protein 3 (JIP3), and HAP1, during retrograde trafficking [181], but the mechanisms for their differential recruitment are unknown. Interestingly, work in PC12 cells indicated that endolysosomes repeatedly contact axonal ER during transit within neurite projections to coordinate exchanges of motor proteins and adaptor proteins that help drive motility [86]. Furthermore, lysosome contacts with ER near the soma appear to be required for lysosomal transport within axons [182], further supporting a regulatory link between ER and the autophagy–lysosome pathway. However, while the relevance of this mechanism to autophagosomes needs to be tested, HTT is also capable of associating with ER [140], making it a promising candidate to explore in mediating the exchange of motor proteins and adaptors between endolysosomes/autophagosomes and the axonal ER. Taken together, HTT appears to play a critical role in coordinating the retrograde movement of autophagosomes and endolysosomes, but further explorations are needed to test if HTT also coordinates the scaffolding of the SNARE fusion machinery responsible for autophagosome fusion with endolysosomes. Moreover, precise markers specific to different endolysosome and autophagosome cargoes which are currently lacking (for e.g., amphisomes versus autophagosomes, catalytically competent endolysosomes,) would aid in identifying the exact steps in the autophagy–lysosome pathway.

### Regulatory mechanisms of neuronal autophagy induction

Autophagy in neurons exhibit unique regulatory characteristics compared to non–neuronal cells, notably showing less susceptibility to mTOR regulation [47]. Neurons rely on highly active constitutive autophagy under basal conditions [8]. However, the upstream pathways that activate ULK1–mediated autophagy in neurons, or potential alternative pathways, remain to be fully understood. Being post–mitotic, neurons likely adapted multiple pathways that can trigger clearance of damaged proteins and organelles in response to intrinsic and extrinsic stressors. Since we do not have a clear picture of the diverse autophagy induction pathways within neurons, it is difficult to understand how these might go awry and contribute to neurodegenerative diseases, including HD. While HTT interacts with ULK1 independent of the mTOR–ULK1 complex in fly and human cells [33,174], whether similar associations also occur in neurons is unclear. Therefore, it is unknown if HTT has a role during neuronal autophagy induction. Loss of HTT reduced autophagy activity in fly neurons [37] as well as mouse embryonic fibroblasts (MEFs), N2a neuroblastoma cells, and striatal cells [33]. However, mice expressing excess normal, non–diseased, wildtype HTT exhibited enhanced autophagy activity [31]. In contrast, other work failed to find HTT co–localising with ATG5 in axons of fly neurons [40]. While it is unclear if the HTT–ULK1 complex functions in ATG5–independent pathways, it is possible that HTT could play a specific role initially prior to autophagosome assembly. Alternatively, certain small molecules can induce autophagy in neurons, providing a clue to the involvement of other kinases in upstream signalling pathways. For example, 10–[4′–(N–diethylamino)butyl]–2–chlorophenoxazin (10–NCP), an AKT inhibitor, has been shown to enhance autophagy in mouse striatal, cortical, and hippocampal neurons [48]. 10–NCP was further shown to enhance the retrograde axonal transport of late endosomes and autophagosomes in cortical neurons expressing mutant human amyloid–beta precursor protein [APP; 183], supporting a link between retrograde axonal transport and autophagy activity. While it is unknown if HTT is involved in this same pathway, work on 10–NCP opens an exciting avenue to explore other kinase pathways that may be involved during autophagy induction. Further, it would also be interesting to explore how the HTT–ULK1 complex changes in the presence or absence of autophagy inducing agents such as 10–NCP to determine if this complex is a requirement for neuronal autophagy activity.

#### Spatial production of autophagosomes within a neuron

**Figure 2.**
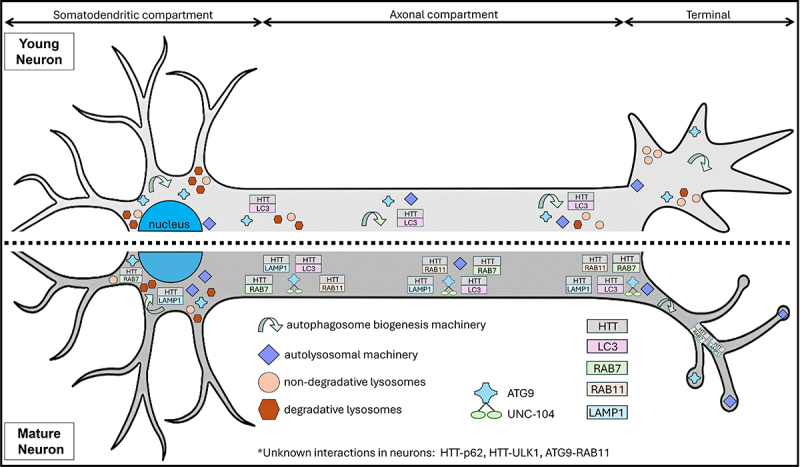
Spatial and temporal distributions of selected machinery in the autophagy–lysosome pathway. In a young neuron with an active growth cone, degradative lysosomes are primarily localised within the somatodendritic compartment, while non–degradative lysosomes are more prevalent in axons and terminals. Autophagosome biogenesis machinery, including ATG9, and autolysosomal components are distributed across the neuronal compartments, though the precise mechanisms of their trafficking remain unclear. In axons, HTT and LC3 co–transport, suggesting a coordinated role in autophagy. In a mature neuron with a fully developed synaptic terminal, both degradative and non–degradative lysosomes are present within the somatodendritic compartment, although their precise distribution in axons and terminals remains less defined. Autophagosome biogenesis machinery is present in the somatodendritic compartment and axon terminals, but their specific distribution and mechanisms of transport within axons are still unclear. In contrast, autolysosomal machinery is distributed throughout the depicted neuronal compartments, yet the mechanisms governing their transport within axons are not fully understood. In mature neurons, ATG9 vesicles are transported within axons by UNC–104, while HTT is observed to move within axons in association with RAB7, LAMP1, LC3, or RAB11.

Due to their complex architecture, neurons are faced with a unique issue since cargo that needs to be degraded can be in distal processes, far from the soma where a lysosomal machinery is predominantly concentrated. This necessitates a highly coordinated and localised generation of autophagosomes within the distal regions of the neuron to ensure efficient degradation of cellular components. Localised autophagosome generation allows for the immediate encapsulation and degradation of cargo, preventing the accumulation of potentially toxic materials like protein aggregates and damaged organelles. ATG9–mediated phagophore assembly has been implicated across a wide range of membranous compartments [184] and ATG9–containing vesicles have been shown to contribute to autophagosome formation [185]. Though ATG9–containing vesicles were identified from subcellular fractionation of rat brain tissue, it remains unclear how ATG9 is trafficked, distributed, and activated within distal axonal processes. ATG 9 was shown to be enriched at nerve terminals, therefore ATG9 must be transported from the soma to the synapse Figure 2, [186]. Vesicular ATG9 was also observed to move anterogradely in C. elegans with RAB3 by the aid of KIF1A/UNC–104 which transports synaptic vesicles [71], and HTT has been observed to facilitate the axonal transport of RAB3–containing vesicles in fly axons [150]; however, it is unknown if this putative HTT–RAB3 vesicle overlaps with ATG9–RAB3 vesicles. Moreover, these ATG9 vesicles may also undergo exo/endocytosis at the pre synapse in response to stimulations, similar to what is observed for Rab11 and HTT containing vesicles [187].

Interestingly, the HTT N–terminus has been proposed to maintain similarity with ATG23 [175], an upstream autophagy protein critical for ATG9 trafficking in yeast [188]. Isolation of ATG9–containing vesicles from rat brain tissue suggests that HTT may be present with these vesicles [186] and a BiolD proteomics analysis of ATG9 interactors in HEK293 cells revealed HTT, HIP1, and 40–kDa huntingtin–associated protein [HAP40; 189]. However, it remains unknown if HTT directly interacts with ATG9 and/or if it regulates the distribution of ATG9–containing vesicles within neurons. Although ATG9 localises with RAB11–containing vesicles in HEK293 cells, and HTT has been shown to mediate the axonal transport of RAB11–containing vesicles in fly axons [149], it is uncertain if the RAB11–ATG9 and RAB11–HTT vesicle complexes are overlapping in the context of autophagy.

Further, the recruitment of ATG9 to ULK1 [190] remains poorly studied in neurons, leaving speculation that perhaps the HTT–ULK1 complex [33,174] is playing a vital role coordinating this recruitment. Indeed, the recruitment of ATG9 to ULK1 has been suggested to be dependent on adaptor protein complex 2 (AP–2) via interactions with TBC1 Domain Family Member 5 [TBC1D5; 191], a major component of clathrin–mediated endocytosis [CME, 192]. In neurons, CME can involve PI4,5P2 [193], huntingtin–interacting protein 1 [HIP1, 194] and dynamin 1 [195]. Independent studies have shown that HTT interacts with all these components [139,196,197] and AP–2 interacts with HIP1 [194,198]. However, it is unknown if HTT plays a role during CME/endocytosis and whether such a role helps coordinate ULK1 and ATG9 in the autophagy–lysosome pathway in neurons and particularly in the distal regions of the axons.

### Temporal regulation of autophagy in an ageing neuron

In humans, neurons project long axons that innervate target cells up to 1 metre away from their cell body. Young neurons, characterised by active growth and high plasticity, exhibit robust axon outgrowth driven by dynamic growth cones at their tips [199]. These growth cones, rich in actin and microtubules, navigate the extracellular environment by responding to various guidance cues, facilitating rapid synaptogenesis. In contrast, mature neurons focus on maintaining established synaptic connections, displaying reduced plasticity and a more stabilised cytoskeleton. This transition involves a shift in molecular composition, with decreased expression of growth–associated proteins and increased stabilisation of the cytoskeleton through neurofilaments and synaptic proteins [200]. The use of adult neurons in culture from mice or rats is challenging due to their inherently poor axon regenerative capacity, influenced by both intrinsic downregulation of growth–promoting genes [201,202]. Consequently, researchers often utilise embryonic neurons from mice or rats for their superior axon outgrowth capabilities [203], despite these cells not fully representing a mature neuronal state. Furthermore, cultured neuronal systems predominantly study actively growing axons with growth cones, focusing on mechanisms of axon guidance and extension, whereas the dynamics of terminally synapsed axons, which involve the maintenance and function of established synaptic connections, are less frequently explored. Additionally, the use of human iPSC–derived neurons poses challenges as they lose the age/maturation characteristics of the individual donor, due to the reprogramming process that reverts fibroblasts to a pluripotent state [204,205]. While ongoing research aims to induce ageing [206] or directly differentiate human fibroblasts into neurons [207], bypassing the need to generate stem cells first, the metrics for accurately modelling aged or mature human neurons in culture remain non–standardised. This highlights the ongoing difficulty in creating in vitro models that accurately reflect the physiological and pathological states of mature human neurons. In contrast, model systems such as Drosophila and C. elegans can help overcome this gap in the field as in vivo models of neurons with long axons with terminal synapses.

Such challenge surfaces when studying proteins such as HTT. Loss of HTT in mice leads to embryonic lethality by embryonic day 7.5 [124,125]. However, conflicting evidence leaves the importance of HTT in adult mice to be controversial. While 35,showed that loss of HTT caused neuronal/axon degeneration and early mortality in 8–month–old mice, 208,found that loss of HTT in adult mice (4– & 8–month–old) exhibited normal lifespans. Reduced HTT in adult mice was shown to cause abnormalities such as increased striatal neuropathy [209]. Similar to HTT, the distinctions between mechanisms driving autophagy in young neurons versus mature neurons has scarcely been examined despite being essential for both neuronal development and neuronal survival/homoeostasis [210]. Interestingly, loss of autophagy was shown to affect lifespan in C. elegans, Drosophila, and mice – with key genes in the autophagy–lysosome pathway being observed to undergo an age–dependent decline in expression. Investigating how ageing and maturation of neurons influence autophagy and the proteins involved, including HTT, is crucial for understanding the distinctive roles these process is affected at different life stages. Elucidating the differences in autophagic mechanisms between young and mature neurons will also allow researchers to gain insights into how to enhance autophagy therapeutically in the context of neurodegeneration, in established neuronal populations.

## Autophagy in HD

**Figure 3.**
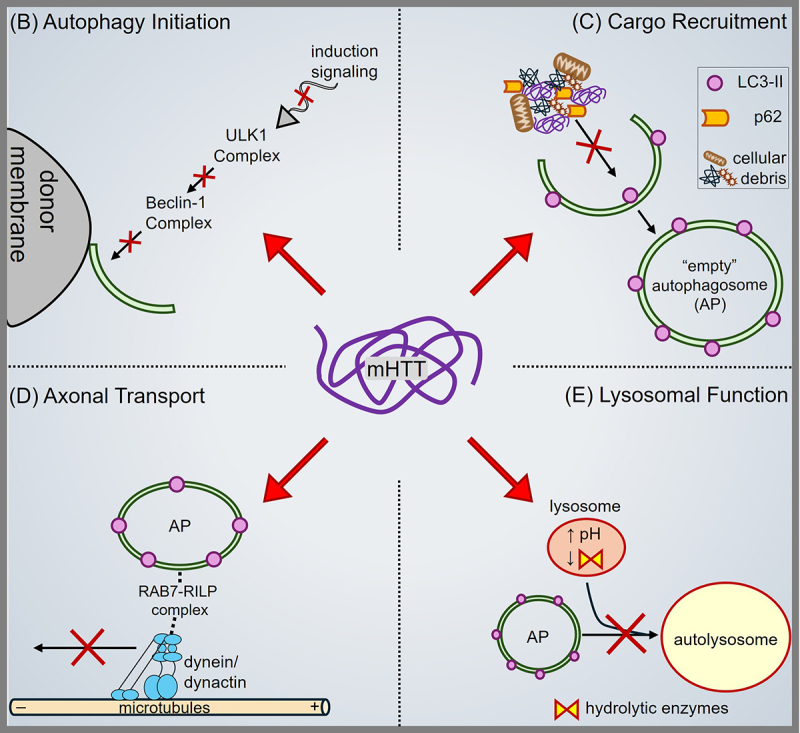
Pathogenic HTT disrupts the Autophagy–Lysosome Pathway in HD. Pathogenic mHTT has been evidenced to cause multi–faceted disruption of the autophagy–lysosome pathway, leading to (A) defective induction signalling for autophagy initiation, (B) disrupted cargo recruitment during autophagosome formation leading to “empty” autophagosomes and accumulations of cellular debris in the cytosol, (C) perturbed axonal transport of autophagy–related compartments such as autophagosomes and lysosomes, and (D) impaired lysosomal function underscored by deficient acidification, reduced abundance of lysosomal hydrolytic enzymes, and improper autophagosome–lysosome fusion.

HD is a devastating, dominantly inherited neurodegenerative disorder that manifests from an N–terminal CAG repeat expansion in the HTT gene. Elucidating mechanisms underlying mHTT–mediated autophagy defects is challenging due to evidence that HD progression derives from gain–of–function [34], loss–of–function [35,36], and/or aberrant polyQ–mediated events. While growing evidence supports a role for normal, wildtype HTT in the autophagy–lysosome pathway [33,39], HD transgenic mice showed autophagosome–like vacuole accumulations prior to detectable disease onset [211], suggesting autophagy defects might underscore HD pathogenesis (Figure 3). Below, we will discuss how pathogenic mHTT can lead to (1) defective autophagy initiation, (2) impaired cargo loading of autophagosomes, (3) perturbed axonal transport of autophagy–lysosome compartments, and (4) disrupted lysosomal functions. This multi–faceted dysregulation of autophagy in HD highlights the need for therapeutic strategies that can restore autophagy activity and enhance clearance of toxic mHTT aggregates, potentially mitigating neuronal loss and disease progression.

### Defective autophagy initiation

Post–Mortem analyses of brains from HD patients revealed accumulations of endolysosomal vesicles [26,27]. However, observations in HD transgenic mice show reduced autophagy–mediated degradation with mHTT expression as indicated by accumulated LC3–II and p62 [30]. Dysregulation of autophagy initiation is one putative mechanism that underscores this decoupling of autophagy initiation from a cell’s degradative capacity (Figure 3A). Work in HEK293 cells suggested that normal, non–pathogenic HTT facilitates mTOR activity by mediating an interaction between mTOR and Rheb, a small G protein, which becomes exacerbated with mHTT expression leading to increased mTOR1 activity [212]. Though we previously discussed the controversial effect of rapamycin on neuronal autophagy, an alternative small molecule inhibitor of mTOR1, CCI–779, was identified to reduce mTOR activity and decrease mHTT aggregates in transgenic HD mice [213]. Parallel to mTOR hyperactivity, AMPK activation has been suggested to be neuroprotective in transgenic HD mice, with potential therapeutic avenues involving the AMPK–activating metformin [214]. However, the role of AMPK in HD pathogenesis is likely complex since later work in HD transgenic mice found that chronic AMPK hyperactivation exacerbated HD pathogenesis with increased mHTT aggregates and increased cell death [215]. Interfering with AMPK pathways via CGS21680, an A(2A) adenosine receptor (A(2A)–R)–selective agonist [216], alleviated the detrimental effects of AMPK hyperactivation in HD transgenic mice [215]. Downstream of autophagy signalling, work in HD transgenic mice suggests differential dysregulation of ULK1 kinase activity with decreased phosphorylation of Beclin–1 and ATG14 [217] but increased phosphorylation of p62 [218]. Moreover, mHTT aggregates sequester Beclin–1 [33]. Since ATG14 phosphorylation [217] and Beclin–1 disinhibition [219] may enhance mHTT clearance, therapeutic efforts targeting activation of ATG14, Beclin–1, and other ULK1 targeting proteins may curtail the complexity surrounding upstream components such as mTOR and AMPK.

### Impaired cargo loading of autophagosomes

While studies in human and rodent HD samples reveal an increased abundance of autophagosomes, these are often functionally impaired, “empty” autophagosomes that lack cargo to be degraded [Figure 3B; 30, 220]. Though biochemical analysis identified that mHTT aberrantly interacts with and sequesters p62 [30], the precise mechanism(s) driving this “fail–to–detect” model remain unclear. In contrast, a “fail–to–engulf” model has recently been proposed in which p62 properly detects cargo, but autophagosomes fail to properly engulf the p62–detected cargo through mechanisms involved during phagophore assembly [221]. Since it is possible that defective autophagosome engulfment and cargo detection can contribute to the formation of “empty” autophagosomes in HD [220], this complicates therapeutic interventions aimed solely at upregulating autophagy and suggests a need for avenues that enhance cargo recruitment/engulfment. A recently developed technology called AUTOTAC (AUTOphagy–Targeting Chimera) uses small peptide agonists that bind and activate p62 to facilitate the selective degradation of cargo through p62–dependent autophagy [222]. Ji et al used AUTOTAC to selectively target mHTT expressed in HeLa cells where they observed increased recruitment of mHTT to LC3–positive compartments as well as increased degradation of mHTT. While AUTOTACs represent a promising platform for targeted protein degradation in HD, critical questions remain regarding their pharmacological properties, catalytic potential, and selectivity. Future studies are needed to optimise their design, address off–target effects, and fully elucidate their mechanisms of action, including the effect of p62 sequestration on target degradation.

### Perturbed axonal transport of autophagy–lysosome compartments

Pathogenic mHTT with aberrant polyQ–expansion exacerbates autophagy dysfunction by disrupting the axonal transport of autophagosomes Figure 3C; [39] and endolysosomes [223]. Previously, we discussed that autophagosomes forming in distal neurite processes require STX17–mediated fusion with RAB7–containing endolysosomes prior to retrograde axonal transport [106]. Recent work in Drosophila identified that HTT and STX17 co–localise on axonal endolysosomes containing RAB7 [40]. One possible mechanism driving impaired axonal transport of autophagosomes is that pathogenic mHTT disrupts STX17–mediated fusion of autophagy–lysosome compartments. Furthermore, mHTT has been shown to aberrantly interact with HAP1 [224], and HTT–HAP1 associations are critical for the retrograde axonal transport of autophagosomes [180,181]. Therefore, it is also possible that mHTT leads to discoordination and improper recruitment of dynein motor proteins and regulators involved in retrograde axonal transport. Similarly, pathogenic mHTT caused a weakened interaction between RILP and dynactin p150Glued, a co–factor protein associated with dynein [225]. Moreover, mHTT also impaired a complex containing OPTN and RAB8 responsible for lysosomal trafficking that resulted in decreased lysosomal activity and a lower lysosomal content of proteases like cathepsin D [226]. Taken together, targeting complexes containing STX17, RILP, or OPTN/RAB8 would be promising candidates to directly alleviate mHTT–mediated transport dysfunction in the autophagy–lysosome pathway.

### Disrupted lysosomal functions

Studies in iPSC–derived neurons from HD patients revealed impaired autophagosome–lysosome fusion and reduced clearance of mHTT aggregates Figure 3D; [28]. Additionally, mHTT can impair lysosomal function by altering lysosomal pH and inhibiting lysosomal enzymes Figure 3D; [29]. Trehalose is a natural disaccharide that stimulates lysosomal biogenesis by causing lysosomal enlargement and low–grade lysosomal stress that leads to activation of TFEB [227]. Trehalose was successful in HD pathogenesis in transgenic HD mice [228]. Since trehalose also disrupts lysosomal acidification [227], there is concern that chronic application of trehalose may lead to unwanted secondary effects in cells less vulnerable to pathogenic mHTT in HD. Complimentary to this approach would be the use of acidifying nanoparticles to restore lysosomal acidification in HD. Acidifying nanoparticles have been shown to traffic to lysosomes and promote acidification in ARPE–19 cells [229], pancreatic β–Cells [230], and dopaminergic neurons from a mouse model of Parkinson’s disease [231]. Perhaps a combination of trehalose and acidifying nanoparticles could provide a dual–targeted approach to improve lysosomal function and mitigate the effects of mHTT aggregation in HD.

Extracellular vesicles have emerged as a compensatory mechanism, facilitating the secretion and removal of mHTT aggregates when the autophagy–lysosome pathway is dysfunctional [232]. The autophagy–lysosome pathway and extracellular vesicles (EVs), including exosomes, exhibit a dynamic interplay critical for maintaining cellular homoeostasis [233]. Notably, mHTT has been detected in EVs isolated from transgenic and knock–in porcine models of HD, as well as in the plasma of HD patients [234]. By encapsulating and transporting mHTT aggregates to extracellular spaces or neighbouring cells, EVs can reduce the intracellular burden of toxic mHTT aggregates. However, this mechanism also carries the risk of contributing to non–cell–autonomous pathology, as the prion–like propagation of mHTT aggregates may exacerbate disease progression through cell–to–cell transmission [235]. Understanding the dual role of EVs in mitigating intracellular toxicity and potentially driving intercellular pathology underscores the therapeutic potential of targeting the autophagy–EV axis in HD. Modulating these pathways may offer innovative strategies to enhance aggregate clearance while limiting their pathological spread, presenting a promising avenue for therapeutic development.

## Concluding remarks

In summary, this review illuminates the intricate relationship between HTT and neuronal autophagy. The emerging consensus is that HTT plays a multifaceted role in this process, influencing various stages from induction to cargo recruitment and transport. HTT’s scaffolding properties enable interactions with key autophagy machinery, including ULK1, p62, HAP1, and molecular motors like kinesin–1 and dynein/dynactin. These interactions likely facilitate the coordination of autophagosome biogenesis, cargo loading, and trafficking (Figure 1), thus influencing neuronal homoeostasis and function. However, the precise mechanisms governing these interactions, and the specific subcellular locations where they occur, are still uncertain and need to be investigated.

Neurons, with their unique challenges in maintaining cellular homoeostasis due to their post–mitotic nature and extensive axonal networks, rely on a finely tuned autophagic system. The distinct regulatory mechanisms of neuronal autophagy induction, spatial production of autophagosomes, and axonal transport mechanisms highlight the specialised nature of this process (Figure 2). Moreover, the temporal regulation of autophagy in ageing neurons underscores the need to differentiate between mechanisms in young, growing neurons versus mature neurons with established synapses (Figure 2). Future studies using diverse model systems, including aged human iPSC–derived neurons and in vivo models such as Drosophila, C. elegans, and mice will be crucial to unravelling these complexities.

The potential therapeutic implications of modulating neuronal autophagy in neurodegenerative diseases such as HD cannot be overstated (Figure 3). A deeper understanding of the precise mechanisms by which HTT influences neuronal autophagy, in conjunction with the development of specific markers for different autophagic compartments, will likely pave the way for targeted interventions aimed at restoring neuronal function and combating the devastating effects seen in HD.

## Abbreviations

Huntingtin: HTT
Huntington’s disease: HD
Mutant huntingtin: mHTT
Mass spectrometry: MS
Yeast two–hybrid: Y2H
Induced pluripotent stem cell: iPSC
Ubiquitin–proteasome system: UPS
Amyotrophic lateral sclerosis: ALS
Parkinson’s disease: PD
Alzheimer’s disease: AD
Mammalian target of rapamycin 1: mTOR
unc–51–like autophagy activating kinase: ULK1
focal adhesion kinase family interacting protein of 200 kDa: FIP200
AuTophaGy–related: ATG
Vacuolar protein sorting: VPS
AMP–activated kinase: AMPK
Glycogen synthase kinase 3β: GSK3β
Protein kinase C α: PKCα
Transcription factor EB: TFEB
phosphatidylinositol 3–phosphate: PI3P
WD repeat domain phosphoinositide–interacting protein: WIPI
Microtubule–associated protein 1A/1B–light chain 3: LC3
Fab–1, YGL023, Vps27, and EEA1: FYVE
Phosphatidylethanolamine: PE
Transmembrane protein 41B: TMEM41B
Endoplasmic reticulum: ER
LC3–interacting region: LIR
Sequestosome 1: SQSTM1
Neighbor of BRCA1: NBR1
Tax1 binding protein 1: TAX1BP1
Nuclear dot protein 52: NDP52
Optineurin: OPTN
PTEN–induced kinase 1: PINK1
NF–kB essential modulator: NEMO
Soluble N–ethylmaleimide–sensitive factor attachment receptor: SNARE
Syntaxin 17: STX17
Syntaxin 7: STX7
Lysosome–associated membrane protein 1: LAMP1
RAB7–interacting lysosome protein: RILP
Homotypic fusion and protein sorting: HOPS
Oxysterol–binding protein–related protein 1L: ORP1L
FYVE and coiled–coil domain–containing protein 1: FYCO1
Huntingtin, Elongator factor3, PR65/A regulatory subunit of PP2A, and Tor1: HEAT
Phosphoinositide: PI
Phosphatidylinositol 4–phosphate: PI4P
Phosphatidylinositol 3,5–bisphosphate: PI3,5P2
Phosphatidylinositol 3,5–bisphosphate: PI4,5P2
Human embryonic kidney 293: HEK293
c–Jun N–terminal kinase–interacting protein 1: JIP1
c–Jun N–terminal kinase–interacting protein 3: JIP3
Huntingtin–associated protein 1: HAP1
Amyloid–beta precursor protein: APP
10–[4′–(*N*–diethylamino)butyl]–2–chlorophenoxazin: 10–NCP
Adaptor protein complex 2: AP–2
TBC1 Domain Family Member 5: TBC1D5
Clathrin–mediated endocytosis: CME
Huntingtin–interacting protein 1: HIP1
40–kDa huntingtin–associated protein: HAP40

## References

[1] Türker F, Cook EK, Margolis SS. The proteasome and its role in the nervous system. Cell Chem Biol. 2021;28(7):903–36. 33905676 10.1016/j.chembiol.2021.04.003 PMC8286317

[2] Winckler B, Faundez V, Maday S, et al. The Endolysosomal system and proteostasis: from development to degeneration. J Neurosci. 2018;38(44):9364–9374. 30381428 10.1523/JNEUROSCI.1665-18.2018 PMC6209849

[3] Liénard C, Pintart A, Bomont P. Neuronal autophagy: regulations and implications in health and disease. Cells. 2024;13(1). doi:10.3390/cells13010103 PMC10778363 38201307

[4] Xie Z, Klionsky DJ. Autophagosome formation: core machinery and adaptations. Nat Cell Biol. 2007;9(10):1102–1109. 17909521 10.1038/ncb1007-1102

[5] Komatsu M, Wang QJ, Holstein GR, et al. Essential role for autophagy protein Atg7 in the maintenance of axonal homeostasis and the prevention of axonal degeneration. Proc Natl Acad Sci U S A. 2007;104(36):14489–14494. 17726112 10.1073/pnas.0701311104 PMC1964831

[6] Hara T, Nakamura K, Matsui M, et al. Suppression of basal autophagy in neural cells causes neurodegenerative disease in mice. Nature. 2006;441(7095):885–889. 16625204 10.1038/nature04724

[7] Komatsu M, Waguri S, Chiba T, et al. Loss of autophagy in the central nervous system causes neurodegeneration in mice. Nature. 2006;441(7095):880–884. 16625205 10.1038/nature04723

[8] Mizushima N, Yamamoto A, Matsui M, et al. In vivo analysis of autophagy in response to nutrient starvation using transgenic mice expressing a fluorescent autophagosome marker. Mol Biol Cell. 2003;15(3):1101–1111. 14699058 10.1091/mbc.E03-09-0704 PMC363084

[9] Lipinski MM, Zheng B, Lu T, et al. Genome–wide analysis reveals mechanisms modulating autophagy in normal brain aging and in Alzheimer’s disease. Proc Natl Acad Sci U S A. 2010;107(32):14164–14169. 20660724 10.1073/pnas.1009485107 PMC2922576

[10] Ishikawa S, Ishikawa F. Proteostasis failure and cellular senescence in long–term cultured postmitotic rat neurons. Aging Cell. 2019;19(1):e13071. 31762159 10.1111/acel.13071 PMC6974705

[11] Moreno–Blas D, Gorostieta–Salas E, Pommer–Alba A, et al. Cortical neurons develop a senescence–like phenotype promoted by dysfunctional autophagy. Aging (Albany NY). 2019;11(16):6175–6198. 31469660 10.18632/aging.102181 PMC6738425

[12] Beckers J, Tharkeshwar AK, Van Damme P. C9orf72 ALS–FTD: recent evidence for dysregulation of the autophagy–lysosome pathway at multiple levels. Autophagy. 2021 Nov;17(11):3306–3322. Epub 2021 Feb 26. PMID: 33632058; PMCID: PMC8632097 33632058 10.1080/15548627.2021.1872189 PMC8632097

[13] Tokuda E, Brännström T, Andersen PM, et al. Low autophagy capacity implicated in motor system vulnerability to mutant superoxide dismutase. Acta Neuropathol Commun. 2016 Jan 25;4(1):6. PMID: 26810478; PMCID: PMC4727314 26810478 10.1186/s40478-016-0274-y PMC4727314

[14] Dehay B, Bové J, Rodríguez–Muela N, et al. Pathogenic lysosomal depletion in Parkinson’s disease. J Neurosci. 2010;30(37):12535–12544. 20844148 10.1523/JNEUROSCI.1920-10.2010 PMC6633458

[15] Hou X, Watzlawik JO, Fiesel FC, et al. Autophagy in Parkinson’s Disease. J Mol Biol. 2020;432(8):2651–2672. 32061929 10.1016/j.jmb.2020.01.037 PMC7211126

[16] Nixon RA, Wegiel J, Kumar A, et al. Extensive involvement of autophagy in Alzheimer disease: an immuno–electron microscopy study. J Neuropathol Exp Neurol. 2005;64(2):113–122. 15751225 10.1093/jnen/64.2.113

[17] Uddin MS, Stachowiak A, Mamun AA, et al. Autophagy and Alzheimer’s Disease: from molecular mechanisms to therapeutic implications. Front Aging Neurosci. 2018;10:4. 10.3389/fnagi.2018.00004 PMC5797541 29441009

[18] Martin DDO, Ladha S, Ehrnhoefer DE, et al. Autophagy in Huntington disease and huntingtin in autophagy. Trends Neurosci. 2014 Jan;38(1):26–35. Epub 2014 Oct 2. PMID: 25282404 25282404 10.1016/j.tins.2014.09.003

[19] Pircs K, Barker RA, Jakobsson J. Hunting out the autophagic problem in Huntington disease. Autophagy. 2022;18(12):3031–3032. 35468035 10.1080/15548627.2022.2069438 PMC9673967

[20] Reiner A, Albin RL, Anderson KD, et al. Differential loss of striatal projection neurons in Huntington disease. Proc Natl Acad Sci U S A. 1988;85(15):5733–5737. 2456581 10.1073/pnas.85.15.5733 PMC281835

[21] Nance MA, Sanders G. Characteristics of individuals with Huntington disease in long–term care. Mov Disord. 1996;11(5):542–548. 8866495 10.1002/mds.870110509

[22] Nance MA. Genetic testing of children at risk for Huntington’s disease. US Huntington disease genetic testing group. Neurology. 1997 Oct;49(4):1048–1053. PMID: 9339688 9339688 10.1212/wnl.49.4.1048

[23] Duyao M, Ambrose C, Myers R, et al. Trinucleotide repeat length instability and age of onset in Huntington’s disease. Nat Genet. 1993;4(4):387–392. 8401587 10.1038/ng0893-387

[24] Semaka A, Kay C, Doty C, et al. CAG size–specific risk estimates for intermediate allele repeat instability in Huntington disease. J Med Genet. 2013;50(10):696–703. 23896435 10.1136/jmedgenet-2013-101796

[25] Genetic Modifiers of Huntington’s Disease (GeM–HD) Consortium. Electronic address: gusella@helix. mgh. harvard.edu, genetic modifiers of Huntington’s Disease (GeM–HD) consortium. CAG repeat not polyglutamine length determines timing of huntington’s disease onset. Cell. 2019;178(4):887–900.e14. 31398342 10.1016/j.cell.2019.06.036 PMC6700281

[26] Sapp E, Schwarz C, Chase K, et al. Huntingtin localization in brains of normal and Huntington’s disease patients. Ann Neurol. 1997;42(4):604–612. 9382472 10.1002/ana.410420411

[27] Tellez–Nagel I, Johnson AB, Terry RD. Studies on brain biopsies of patients with Huntington’s chorea. J Neuropathol Exp Neurol. 1974;33(2):308–332. 4150800 10.1097/00005072-197404000-00008

[28] Pircs K, Drouin–Ouellet J, Horváth V, et al. Distinct subcellular autophagy impairments in induced neurons from patients with Huntington’s disease. Brain. 2022;145(9):3035–3057. 34936701 10.1093/brain/awab473 PMC9473361

[29] Kegel KB, Kim M, Sapp E, et al. Huntingtin expression stimulates endosomal–lysosomal activity, endosome tubulation, and autophagy. J Neurosci. 2000;20(19):7268–7278. 11007884 10.1523/JNEUROSCI.20-19-07268.2000 PMC6772788

[30] Martinez–Vicente M, Talloczy Z, Wong E, et al. Cargo recognition failure is responsible for inefficient autophagy in Huntington’s disease. Nat Neurosci. 2010;13(5):567–576. 20383138 10.1038/nn.2528 PMC2860687

[31] Zheng S, Clabough EBD, Sarkar S, et al. Deletion of the huntingtin polyglutamine stretch enhances neuronal autophagy and longevity in mice. PLoS Genet. 2010;6(2):e1000838. 20140187 10.1371/journal.pgen.1000838 PMC2816686

[32] Qin ZH, Wang Y, Kegel KB, et al. Autophagy regulates the processing of amino terminal huntingtin fragments. Hum Mol Genet. 2003;12(24):3231–3244. 14570716 10.1093/hmg/ddg346

[33] Rui YN, Xu Z, Patel B, et al. Huntingtin functions as a scaffold for selective macroautophagy. Nat Cell Biol. 2015;17(3):262–275. 25686248 10.1038/ncb3101 PMC4344873

[34] Aronin N, Chase K, Young C, et al. CAG expansion affects the expression of mutant Huntingtin in the Huntington’s disease brain. Neuron. 1995;15(5):1193–1201. 7576661 10.1016/0896-6273(95)90106-x

[35] Dragatsis I, Levine MS, Zeitlin S. Inactivation of Hdh in the brain and testis results in progressive neurodegeneration and sterility in mice. Nat Genet. 2000 Nov;26(3):6926. PMID: 11062468 10.1038/81593 11062468

[36] Schulte J, Littleton JT. The biological function of the Huntingtin protein and its relevance to Huntington’s Disease pathology. Curr Trends Neurol. 2011;5:65–78 22180703 PMC3237673

[37] Ochaba J, Lukacsovich T, Csikos G, et al. Potential function for the Huntingtin protein as a scaffold for selective autophagy. Proc Natl Acad Sci U S A. 2014;111(47):16889–16894. 25385587 10.1073/pnas.1420103111 PMC4250109

[38] Martin DDO, Heit RJ, Yap MC, et al. Identification of a post–translationally myristoylated autophagy–inducing domain released by caspase cleavage of huntingtin. Hum Mol Genet. 2014;23(12):3166–3179. 24459296 10.1093/hmg/ddu027 PMC4030772

[39] Wong YC, Holzbaur ELF. Optineurin is an autophagy receptor for damaged mitochondria in parkin–mediated mitophagy that is disrupted by an ALS–linked mutation. Proc Natl Acad Sci U S A. 2014;111(42):E4439–48. 25294927 10.1073/pnas.1405752111 PMC4210283

[40] Krzystek TJ, White JA, Rathnayake R, et al. HTT (huntingtin) and RAB7 co–migrate retrogradely on a signaling LAMP1–containing late endosome during axonal injury. Autophagy. 2022;19(4):1199–1220. 36048753 10.1080/15548627.2022.2119351 PMC10012955

[41] Kim J, Kundu M, Viollet B, et al. AMPK and mTOR regulate autophagy through direct phosphorylation of Ulk1. Nat Cell Biol. 2011;13(2):132–141. 21258367 10.1038/ncb2152 PMC3987946

[42] Shang L, Chen S, Du F, et al. Nutrient starvation elicits an acute autophagic response mediated by Ulk1 dephosphorylation and its subsequent dissociation from AMPK. Proc Natl Acad Sci U S A. 2011;108(12):4788–4793. 21383122 10.1073/pnas.1100844108 PMC3064373

[43] Puente C, Hendrickson RC, Jiang X. Nutrient–regulated phosphorylation of ATG13 inhibits starvation–induced autophagy. J Biol Chem. 2016;291(11):6026–6035. 26801615 10.1074/jbc.M115.689646 PMC4786734

[44] Alirezaei M, Kemball CC, Flynn CT, et al. Short–term fasting induces profound neuronal autophagy. Autophagy. 2010;6(6):702–710. 20534972 10.4161/auto.6.6.12376 PMC3106288

[45] Young JE, Martinez RA, La Spada AR. Nutrient deprivation induces neuronal autophagy and implicates reduced insulin signaling in neuroprotective autophagy activation. J Biol Chem. 2009;284(4):2363–2373. 19017649 10.1074/jbc.M806088200 PMC2629092

[46] Ehrnhoefer DE, Martin DDO, Schmidt ME, et al. Preventing mutant huntingtin proteolysis and intermittent fasting promote autophagy in models of Huntington disease. Acta Neuropathol Commun. 2018;6(1):16. 29510748 10.1186/s40478-018-0518-0 PMC5839066

[47] Boland B, Kumar A, Lee S, et al. Autophagy induction and autophagosome clearance in neurons: relationship to autophagic pathology in Alzheimer’s disease. J Neurosci. 2008 Jul 2;28(27):6926–6937. PMID: 18596167; PMCID: PMC2676733 18596167 10.1523/JNEUROSCI.0800-08.2008 PMC2676733

[48] Tsvetkov AS, Miller J, Arrasate M, et al. A small–molecule scaffold induces autophagy in primary neurons and protects against toxicity in a Huntington disease model. Proc Natl Acad Sci U S A. 2010 Sep 28;107(39):16982–16987. Epub 2010 Sep 10. PMID: 20833817; PMCID: PMC2947884 20833817 10.1073/pnas.1004498107 PMC2947884

[49] Tang G, Gudsnuk K, Kuo SH, et al. Loss of mTOR–dependent macroautophagy causes autistic–like synaptic pruning deficits. Neuron. 2014;83(5):1131–1143. 25155956 10.1016/j.neuron.2014.07.040 PMC4159743

[50] Kuijpers M, Kochlamazashvili G, Stumpf A, et al. Neuronal autophagy regulates presynaptic neurotransmission by controlling the axonal endoplasmic reticulum. Neuron. 2021 Jan 20;109(2):299–313.e9. Epub 2020 Nov 5. Erratum in: Neuron. 2022 Feb 16;110(4):734. doi: 10.1016/j.neuron.2022.01.029. PMID: 33157003; PMCID: PMC7837115 33157003 PMC7837115

[51] Domise M, Sauvé F, Didier S, et al. Neuronal AMP–activated protein kinase hyper–activation induces synaptic loss by an autophagy–mediated process. Cell Death Dis. 2019;10(3):221. 30833547 10.1038/s41419-019-1464-x PMC6399353

[52] Ryu HY, Kim LE, Jeong H, et al. GSK3B induces autophagy by phosphorylating ULK1. Exp Mol Med. 2021;53(3):369–383. 33654220 10.1038/s12276-021-00570-6 PMC8080724

[53] Wang C, Wang H, Zhang D, et al. Phosphorylation of ULK1 affects autophagosome fusion and links chaperone–mediated autophagy to macroautophagy. Nat Commun. 2018;9(1):3492. 30154410 10.1038/s41467-018-05449-1 PMC6113293

[54] Chae CW, Yoon JH, Lim JR, et al. TRIM16–mediated lysophagy suppresses high–glucose–accumulated neuronal Aβ. Autophagy. 2023 Oct;19(10):2752–2768. Epub 2023 Jul 4. PMID: 37357416; PMCID: PMC10472864 37357416 10.1080/15548627.2023.2229659 PMC10472864

[55] Itakura E, Kishi C, Inoue K, et al. Beclin 1 forms two distinct phosphatidylinositol 3–kinase complexes with mammalian Atg14 and UVRAG. Mol Biol Cell. 2008 Dec;19(12):5360–5372. Epub 2008 Oct 8. PMID: 18843052; PMCID: PMC2592660 18843052 10.1091/mbc.E08-01-0080 PMC2592660

[56] Kihara A, Noda T, Ishihara N, et al. Two distinct Vps34 phosphatidylinositol 3–kinase complexes function in autophagy and carboxypeptidase Y sorting in *Saccharomyces cerevisiae*. J Cell Biol. 2001;152(3):519–530. 11157979 10.1083/jcb.152.3.519 PMC2196002

[57] Russell RC, Tian Y, Yuan H, et al. ULK1 induces autophagy by phosphorylating Beclin–1 and activating VPS34 lipid kinase. Nat Cell Biol. 2013;15(7):741–750. 23685627 10.1038/ncb2757 PMC3885611

[58] Blommaart EF, Krause U, Schellens JP, et al. The phosphatidylinositol 3–kinase inhibitors wortmannin and LY294002 inhibit autophagy in isolated rat hepatocytes. Eur J Biochem. 1997;243(1–2):240–246. 9030745 10.1111/j.1432-1033.1997.0240a.x

[59] Choi S, Houdek X, Anderson RA. Phosphoinositide 3–kinase pathways and autophagy require phosphatidylinositol phosphate kinases. Adv Biol Regul. 2018;68:31–38. 29472147 10.1016/j.jbior.2018.02.003 PMC5955796

[60] Stenmark H, Aasland R, Driscoll PC. The phosphatidylinositol 3–phosphate–binding FYVE finger. FEBS Lett. 2002 Feb 20;513(1):77–84. PMID: 11911884 11911884 10.1016/s0014-5793(01)03308-7

[61] Ichimura Y, Kirisako T, Takao T, et al. A ubiquitin–like system mediates protein lipidation. Nature. 2000;408(6811):488–492. 11100732 10.1038/35044114

[62] Mizushima N, Noda T, Yoshimori T, et al. A protein conjugation system essential for autophagy. Nature. 1998;395(6700):6700. 10.1038/26506 9759731

[63] Mizushima N, Yamamoto A, Hatano M, et al. Dissection of autophagosome formation using Apg5–deficient mouse embryonic stem cells. J Cell Biol. 2001;152(4):657–668. 11266458 10.1083/jcb.152.4.657 PMC2195787

[64] Maday S, Holzbaur ELF. Autophagosome biogenesis in primary neurons follows an ordered and spatially regulated pathway. Dev Cell. 2014;30(1):71–85. 25026034 10.1016/j.devcel.2014.06.001 PMC4109719

[65] Glick D, Barth S, Macleod KF. Autophagy: cellular and molecular mechanisms. J Pathol. 2010;221(1):3–12. 20225336 10.1002/path.2697 PMC2990190

[66] Moretti F, Bergman P, Dodgson S, et al. TMEM41B is a novel regulator of autophagy and lipid mobilization. EMBO Rep. 2018;19(9). 10.15252/embr.201845889 PMC6123663 30126924

[67] Karanasios E, Walker SA, Okkenhaug H, et al. Autophagy initiation by ULK complex assembly on ER tubulovesicular regions marked by ATG9 vesicles. Nat Commun. 2016;7(1):12420. 27510922 10.1038/ncomms12420 PMC4987534

[68] Reggiori F, Shintani T, Nair U, et al. Atg9 cycles between mitochondria and the pre–autophagosomal structure in yeasts. Autophagy. 2005;1(2):101–109. 16874040 10.4161/auto.1.2.1840 PMC1762033

[69] Imai K, Hao F, Fujita N, et al. Atg9A trafficking through the recycling endosomes is required for autophagosome formation. J Cell Sci. 2016;129(20):3781–3791. 27587839 10.1242/jcs.196196

[70] Luo Q, Liu Q, Cheng H, et al. Nondegradable ubiquitinated ATG9A organizes Golgi integrity and dynamics upon stresses. Cell Rep. 2022;40(7):111195. 35977480 10.1016/j.celrep.2022.111195

[71] Stavoe AKH, Hill SE, Hall DH, et al. KIF1A/UNC–104 transports ATG–9 to regulate neurodevelopment and autophagy at synapses. Dev Cell. 2016;38(2):171–185. 27396362 10.1016/j.devcel.2016.06.012 PMC4961624

[72] Hollenbeck PJ. Products of endocytosis and autophagy are retrieved from axons by regulated retrograde organelle transport. J Cell Biol. 1993;121(2):305–315. 7682217 10.1083/jcb.121.2.305 PMC2200099

[73] Hailey DW, Rambold AS, Satpute–Krishnan P, et al. Mitochondria supply membranes for autophagosome biogenesis during starvation. Cell. 2010;141(4):656–667. 20478256 10.1016/j.cell.2010.04.009 PMC3059894

[74] Hamasaki M, Furuta N, Matsuda A, et al. Autophagosomes form at ER–mitochondria contact sites. Nature. 2013 Mar 21;495(7441):389–393. Epub 2013 Mar 3. PMID: 23455425 23455425 10.1038/nature11910

[75] Hayashi–Nishino M, Fujita N, Noda T, et al. A subdomain of the endoplasmic reticulum forms a cradle for autophagosome formation. Nat Cell Biol. 2009;11(12):1433–1437. 19898463 10.1038/ncb1991

[76] van der Vaart A, Griffith J, Reggiori F. Exit from the Golgi is required for the expansion of the autophagosomal phagophore in yeast *Saccharomyces cerevisiae*. Mol Biol Cell. 2010;21(13):2270–2284. 20444982 10.1091/mbc.E09-04-0345 PMC2893990

[77] Puri C, Vicinanza M, Ashkenazi A, et al. The RAB11A–positive compartment is a primary platform for autophagosome assembly mediated by WIPI2 recognition of PI3P–RAB11A. Dev Cell. 2018;45(1):114–131.e8. 29634932 10.1016/j.devcel.2018.03.008 PMC5896254

[78] Tsukita S, Ishikawa H. Three–dimensional distribution of smooth endoplasmic reticulum in myelinated axons. J Electron Microsc (Tokyo). 1976;25(3)141–149. 1025229

[79] Villegas R, Martinez NW, Lillo J, et al. Calcium release from intra–axonal endoplasmic reticulum leads to axon degeneration through mitochondrial dysfunction. J Neurosci. 2014;34(21):7179–7189. 24849352 10.1523/JNEUROSCI.4784-13.2014 PMC4028495

[80] Wu Y, Whiteus C, Xu CS, et al. Contacts between the endoplasmic reticulum and other membranes in neurons. Proc Natl Acad Sci U S A. 2017;114(24):E4859–E4867. 28559323 10.1073/pnas.1701078114 PMC5474793

[81] González C, Cánovas J, Fresno J, et al. Axons provide the secretory machinery for trafficking of voltage–gated sodium channels in peripheral nerve. Proc Natl Acad Sci U S A. 2016;113(7):1823–1828. 26839409 10.1073/pnas.1514943113 PMC4763731

[82] Wang J, Fourriere L, Gleeson PA. Local secretory trafficking pathways in neurons and the role of dendritic Golgi outposts in different cell models. Front Mol Neurosci. 2020;13:597391. 33324160 10.3389/fnmol.2020.597391 PMC7726432

[83] Oliva MK, Pérez–Moreno JJ, O’Shaughnessy J, et al., Endoplasmic reticulum lumenal indicators in *Drosophila* reveal effects of HSP–related mutations on endoplasmic reticulum calcium dynamics. Front Neurosci. 2020 Aug 10; 14:816. PMID: 32903680; PMCID: PMC7438849 32903680 10.3389/fnins.2020.00816 PMC7438849

[84] Yalçın B, Zhao L, Stofanko M, et al. Modeling of axonal endoplasmic reticulum network by spastic paraplegia proteins. Elife. 2017;6:e23882. Published 2017 Jul 25 28742022 10.7554/eLife.23882 PMC5576921

[85] O’Sullivan NC, Jahn TR, Reid E, et al. Reticulon–like–1, the Drosophila orthologue of the hereditary spastic paraplegia gene reticulon 2, is required for organization of endoplasmic reticulum and of distal motor axons. Hum Mol Genet. 2012 Aug 1;21(15):3356–3365. Epub 2012 Apr 27. PMID: 22543973; PMCID: PMC3392112 22543973 10.1093/hmg/dds167 PMC3392112

[86] Raiborg C, Wenzel EM, Stenmark H. ER –endosome contact sites: molecular compositions and functions. EMBO J. 2015 Jul 14;34(14):1848–1858. Epub 2015 Jun 3. PMID: 26041457; PMCID: PMC4547891 26041457 10.15252/embj.201591481 PMC4547891

[87] Arantes RME, Andrews NW. A role for synaptotagmin VII–regulated exocytosis of lysosomes in neurite outgrowth from primary sympathetic neurons. J Neurosci. 2006;26(17):4630–4637. 16641243 10.1523/JNEUROSCI.0009-06.2006 PMC6674075

[88] Pfenninger KH. Plasma membrane expansion: a neuron’s Herculean task. Nat Rev Neurosci. 2009;10(4):251–261. 19259102 10.1038/nrn2593

[89] Anding AL, Baehrecke EH. Cleaning house: selective autophagy of organelles. Dev Cell. 2017;41(1):10–22. 28399394 10.1016/j.devcel.2017.02.016 PMC5395098

[90] Adriaenssens E, Ferrari L, Martens S. Orchestration of selective autophagy by cargo receptors. Curr Biol. 2022;32(24):R1357–R1371. 36538890 10.1016/j.cub.2022.11.002

[91] Bjørkøy G, Lamark T, Brech A, et al. p62/SQSTM1 forms protein aggregates degraded by autophagy and has a protective effect on huntingtin–induced cell death. J Cell Biol. 2005;171(4):603–614. 16286508 10.1083/jcb.200507002 PMC2171557

[92] Bjørkøy G, Lamark T, Johansen T. p62/SQSTM1: a missing link between protein aggregates and the autophagy machinery. Autophagy. 2006;2(2):138–139. 16874037 10.4161/auto.2.2.2405

[93] Kirkin V, Lamark T, Sou YS, et al. A role for NBR1 in autophagosomal degradation of ubiquitinated substrates. Mol Cell. 2009;33(4):505–516. 19250911 10.1016/j.molcel.2009.01.020

[94] Newman AC, Scholefield CL, Kemp AJ, et al. TBK1 kinase addiction in lung cancer cells is mediated via autophagy of Tax1bp1/Ndp52 and non–canonical NF–κB signalling. PLoS One. 2012;7(11):e50672. 23209807 10.1371/journal.pone.0050672 PMC3510188

[95] Ravenhill BJ, Boyle KB, von Muhlinen N, et al. The cargo receptor NDP52 initiates selective autophagy by recruiting the ULK complex to cytosol–invading bacteria. Mol Cell. 2019;74(2):320–329.e6. 30853402 10.1016/j.molcel.2019.01.041 PMC6477152

[96] Ma S, Attarwala IY, Xie XQ. SQSTM1/p62: a potential target for Neurodegenerative Disease. ACS Chem Neurosci. 2019;10(5):2094–2114. 30657305 10.1021/acschemneuro.8b00516 PMC6712989

[97] Renton AE, Majounie E, Waite A, et al. A hexanucleotide repeat expansion in C9ORF72 is the cause of chromosome 9p21–linked ALS–FTD. Neuron. 2011;72(2):257–268. 21944779 10.1016/j.neuron.2011.09.010 PMC3200438

[98] Chitiprolu M, Jagow C, Tremblay V, et al. A complex of C9ORF72 and p62 uses arginine methylation to eliminate stress granules by autophagy. Nat Commun. 2018;9(1):2794. 30022074 10.1038/s41467-018-05273-7 PMC6052026

[99] Lazarou M, Sliter DA, Kane LA, et al. The ubiquitin kinase PINK1 recruits autophagy receptors to induce mitophagy. Nature. 2015 Aug 20;524(7565):309–314. Epub 2015 Aug 12. PMID: 26266977; PMCID: PMC5018156 26266977 10.1038/nature14893 PMC5018156

[100] Harding O, Holzer E, Riley JF, et al. Damaged mitochondria recruit the effector NEMO to activate NF–κB signaling. Mol Cell. 2023;83(17):3188–3204.e7. 37683611 10.1016/j.molcel.2023.08.005 PMC10510730

[101] Narendra D, Kane LA, Hauser DN, et al. p62/SQSTM1 is required for Parkin–induced mitochondrial clustering but not mitophagy; VDAC1 is dispensable for both. Autophagy. 2010;6(8):1090–1106. 20890124 10.4161/auto.6.8.13426 PMC3359490

[102] Berg TO, Fengsrud M, Strømhaug PE, et al. Isolation and characterization of rat liver amphisomes. Evidence for fusion of autophagosomes with both early and late endosomes. J Biol Chem. 1998;273(34):21883–21892. 9705327 10.1074/jbc.273.34.21883

[103] Jahn R, Scheller RH. SNAREs–engines for membrane fusion. Nat Rev Mol Cell Biol. 2006;7(9):631–643. 16912714 10.1038/nrm2002

[104] Itakura E, Kishi–Itakura C, Mizushima N. The hairpin–type tail–anchored SNARE syntaxin 17 targets to autophagosomes for fusion with endosomes/lysosomes. Cell. 2012;151(6):1256–1269. 23217709 10.1016/j.cell.2012.11.001

[105] Matsui T, Jiang P, Nakano S, et al. Autophagosomal YKT6 is required for fusion with lysosomes independently of syntaxin 17. J Cell Biol. 2018;217(8):2633–2645. 29789439 10.1083/jcb.201712058 PMC6080929

[106] Cheng XT, Zhou B, Lin MY, et al. Axonal autophagosomes recruit dynein for retrograde transport through fusion with late endosomes. J Cell Biol. 2015;209(3):377–386. 25940348 10.1083/jcb.201412046 PMC4427784

[107] Maday S, Holzbaur EL. Compartment–specific regulation of autophagy in primary neurons. J Neurosci. 2016 Jun 1;36(22):5933–5945. PMID: 27251616; PMCID: PMC4887563 27251616 10.1523/JNEUROSCI.4401-15.2016 PMC4887563

[108] Cheng XT, Xie YX, Zhou B, et al. Characterization of LAMP1–labeled nondegradative lysosomal and endocytic compartments in neurons. J Cell Biol. 2018;217(9):3127–3139. 29695488 10.1083/jcb.201711083 PMC6123004

[109] Yap CC, Digilio L, McMahon LP, et al. Degradation of dendritic cargos requires Rab7–dependent transport to somatic lysosomes. J Cell Biol. 2018;217(9):3141–3159. 29907658 10.1083/jcb.201711039 PMC6122995

[110] Farfel–Becker T, Roney JC, Cheng XT, et al. Neuronal soma–derived degradative lysosomes are continuously delivered to distal axons to maintain local degradation capacity. Cell Rep. 2019 Jul 2;28(1):51–64.e4. PMID: 31269450; PMCID: PMC6696943 31269450 10.1016/j.celrep.2019.06.013 PMC6696943

[111] Klionsky DJ, Abdel–Aziz AK, Abdelfatah S, et al. Guidelines for the use and interpretation of assays for monitoring autophagy (4th edition)(1). Autophagy. 2021;17(1):1–382. 33634751 10.1080/15548627.2020.1797280 PMC7996087

[112] Chavrier P, Parton RG, Hauri HP, et al. Localization of low molecular weight GTP binding proteins to exocytic and endocytic compartments. Cell. 1990;62(2):317–329. 2115402 10.1016/0092-8674(90)90369-p

[113] Feng Y, Press B, Wandinger–Ness A. Rab 7: an important regulator of late endocytic membrane traffic. J Cell Biol. 1995;131(6 Pt 1):1435–1452. 8522602 10.1083/jcb.131.6.1435 PMC2120682

[114] Cantalupo G, Alifano P, Roberti V, et al. Rab–interacting lysosomal protein (RILP): the Rab7 effector required for transport to lysosomes. EMBO J. 2001;20(4):683–693. 11179213 10.1093/emboj/20.4.683 PMC145419

[115] Jordens I, Fernandez–Borja M, Marsman M, et al. The Rab7 effector protein RILP controls lysosomal transport by inducing the recruitment of dynein–dynactin motors. Curr Biol. 2001;11(21):1680–1685. 11696325 10.1016/s0960-9822(01)00531-0

[116] Lin X, Yang T, Wang S, et al. RILP interacts with HOPS complex via VPS41 subunit to regulate endocytic trafficking. Sci Rep. 2014;4(1):7282. 25445562 10.1038/srep07282 PMC4250914

[117] Johansson M, Rocha N, Zwart W, et al. Activation of endosomal dynein motors by stepwise assembly of Rab7–RILP–p150Glued, ORP1L, and the receptor betalll spectrin. J Cell Biol. 2007;176(4):459–471. 17283181 10.1083/jcb.200606077 PMC2063981

[118] Johansson M, Olkkonen VM. Assays for interaction between Rab7 and oxysterol binding protein related protein 1L (ORP1L). Methods Enzymol. 2005;403:743–758. 16473636 10.1016/S0076-6879(05)03065-X

[119] Ma X, Liu K, Li J, et al. A non–canonical GTPase interaction enables ORP1L–Rab7–RILP complex formation and late endosome positioning. J Biol Chem. 2018;293(36):14155–14164. 30012887 10.1074/jbc.RA118.001854 PMC6130934

[120] Pankiv S, Alemu EA, Brech A, et al. FYCO1 is a Rab7 effector that binds to LC3 and PI3P to mediate microtubule plus end–directed vesicle transport. J Cell Biol. 2010;188(2):253–269. 20100911 10.1083/jcb.200907015 PMC2812517

[121] Li SH, Schilling G, Young WS 3rd, et al. Huntington’s disease gene (IT15) is widely expressed in human and rat tissues. Neuron. 1993;11(5):985–993. 8240819 10.1016/0896-6273(93)90127-d

[122] Strong TV, Tagle DA, Valdes JM, et al. Widespread expression of the human and rat Huntington’s disease gene in brain and nonneural tissues. Nat Genet. 1993;5(3):259–265. 8275091 10.1038/ng1193-259

[123] The Huntington’s Disease Collaborative Research Group. A novel gene containing a trinucleotide repeat that is expanded and unstable on Huntington’s disease chromosomes. Cell. 1993;72(6):971–983. 8458085 10.1016/0092-8674(93)90585-e

[124] Duyao MP, Auerbach AB, Ryan A, et al. Inactivation of the mouse Huntington’s disease gene homolog Hdh. Science. 1995;269(5222):407–410. 7618107 10.1126/science.7618107

[125] Nasir J, Floresco SB, O’Kusky JR, et al. Targeted disruption of the Huntington’s disease gene results in embryonic lethality and behavioral and morphological changes in heterozygotes. Cell. 1995;81(5):811–823. 7774020 10.1016/0092-8674(95)90542-1

[126] Li W, Serpell LC, Carter WJ, et al. Expression and characterization of full–length human huntingtin, an elongated HEAT repeat protein. J Biol Chem. 2006;281(23):15916–15922. 16595690 10.1074/jbc.M511007200

[127] Andrade MA, Bork P. HEAT repeats in the Huntington’s disease protein. Nat Genet. 1995;11(2):115–116. 7550332 10.1038/ng1095-115

[128] Kobe B, Gleichmann T, Horne J, et al. Turn up the HEAT. Structure. 1999;7(5):R91–R97. 10378263 10.1016/s0969-2126(99)80060-4

[129] Takano H, Gusella JF. The predominantly HEAT–like motif structure of huntingtin and its association and coincident nuclear entry with dorsal, an NF–kB/Rel/dorsal family transcription factor. BMC Neurosci. 2002;3(1):15. 12379151 10.1186/1471-2202-3-15 PMC137586

[130] Saudou F, Humbert S. The biology of huntingtin. Neuron. 2016;89(5):910–926. 26938440 10.1016/j.neuron.2016.02.003

[131] Goehler H, Lalowski M, Stelzl U, et al. A protein interaction network links GIT1, an enhancer of huntingtin aggregation, to Huntington’s disease. Mol Cell. 2004;15(6):853–865. 15383276 10.1016/j.molcel.2004.09.016

[132] Kaltenbach LS, Romero E, Becklin RR, et al. Huntingtin interacting proteins are genetic modifiers of neurodegeneration. PLoS Genet. 2007;3(5):e82. 17500595 10.1371/journal.pgen.0030082 PMC1866352

[133] Culver BP, Savas JN, Park SK, et al. Proteomic analysis of wild–type and mutant huntingtin–associated proteins in mouse brains identifies unique interactions and involvement in protein synthesis. J Biol Chem. 2012;287(26):21599–21614. 22556411 10.1074/jbc.M112.359307 PMC3381125

[134] Ratovitski T, Chighladze E, Arbez N, et al. Huntingtin protein interactions altered by polyglutamine expansion as determined by quantitative proteomic analysis. Cell Cycle. 2012;11(10):2006–2021. 22580459 10.4161/cc.20423 PMC3359124

[135] Shirasaki DI, Greiner ER, Al–Ramahi I, et al. Network organization of the huntingtin proteomic interactome in mammalian brain. Neuron. 2012;75(1):41–57. 22794259 10.1016/j.neuron.2012.05.024 PMC3432264

[136] DiFiglia M, Sapp E, Chase K, et al. Huntingtin is a cytoplasmic protein associated with vesicles in human and rat brain neurons. Neuron. 1995;14(5):1075–1081. 7748555 10.1016/0896-6273(95)90346-1

[137] Chaibva M, Gao X, Jain P, et al. Sphingomyelin and GM1 influence huntingtin binding to, disruption of, and aggregation on lipid membranes. ACS Omega. 2018;3(1):273–285. 29399649 10.1021/acsomega.7b01472 PMC5793032

[138] Kegel KB, Sapp E, Alexander J, et al. Polyglutamine expansion in huntingtin alters its interaction with phospholipids. J Neurochem. 2009;110(5):1585–1597. 19566678 10.1111/j.1471-4159.2009.06255.x

[139] Kegel KB, Sapp E, Yoder J, et al. Huntingtin associates with acidic phospholipids at the plasma membrane. J Biol Chem. 2005;280(43):36464–36473. 16085648 10.1074/jbc.M503672200

[140] Atwal RS, Xia J, Pinchev D, et al. Huntingtin has a membrane association signal that can modulate huntingtin aggregation, nuclear entry and toxicity. Hum Mol Genet. 2007;16(21):2600–2615. 17704510 10.1093/hmg/ddm217

[141] Kim MW, Chelliah Y, Kim SW, et al. Secondary structure of Huntingtin amino–terminal region. Structure. 2009;17(9):1205–1212. 19748341 10.1016/j.str.2009.08.002 PMC2863341

[142] Michalek M, Salnikov ES, Werten S, et al. Membrane interactions of the amphipathic amino terminus of huntingtin. Biochemistry. 2013 Feb 5;52(5):847–858. Epub 2013 Jan 28. PMID: 23305455 23305455 10.1021/bi301325q

[143] Lo Sardo V, Zuccato C, Gaudenzi G, et al. An evolutionary recent neuroepithelial cell adhesion function of huntingtin implicates ADAM10–Ncadherin. Nat Neurosci. 2012;15(5):713–721. 22466506 10.1038/nn.3080

[144] McAdam RL, Morton A, Gordon SL, et al. Loss of huntingtin function slows synaptic vesicle endocytosis in striatal neurons from the htt(Q140/Q140) mouse model of Huntington’s disease. Neurobiol Dis. 2019;134:104637. 31614197 10.1016/j.nbd.2019.104637

[145] McKinstry SU, Karadeniz YB, Worthington AK, et al. Huntingtin is required for normal excitatory synapse development in cortical and striatal circuits. J Neurosci. 2014;34(28):9455–9472. 25009276 10.1523/JNEUROSCI.4699-13.2014 PMC4087216

[146] Brandstaetter H, Kruppa AJ, Buss F. Huntingtin is required for ER–to–Golgi transport and for secretory vesicle fusion at the plasma membrane. Dis Model Mech. 2014;7(12):1335–1340. 25368120 10.1242/dmm.017368 PMC4257002

[147] Gauthier LR, Charrin BC, Borrell–Pagès M, et al. Huntingtin controls neurotrophic support and survival of neurons by enhancing BDNF vesicular transport along microtubules. Cell. 2004;118(1):127–138. 15242649 10.1016/j.cell.2004.06.018

[148] Gunawardena S, Her LS, Brusch RG, et al. Disruption of axonal transport by loss of huntingtin or expression of pathogenic polyQ proteins in Drosophila. Neuron. 2003;40(1):25–40. 14527431 10.1016/s0896-6273(03)00594-4

[149] Power D, Srinivasan S, Gunawardena S. In–vivo evidence for the disruption of Rab11 vesicle transport by loss of huntingtin. Neuroreport. 2012;23(16):970–977. 23032403 10.1097/WNR.0b013e328359d990

[150] White JA 2nd, Anderson E, Zimmerman K, et al. Huntingtin differentially regulates the axonal transport of a sub–set of Rab–containing vesicles in vivo. Hum Mol Genet. 2015;24(25):7182–7195. 26450517 10.1093/hmg/ddv415 PMC4664163

[151] White JA 2nd, Krzystek TJ, Hoffmar–Glennon H, et al. Excess Rab4 rescues synaptic and behavioral dysfunction caused by defective HTT–Rab4 axonal transport in Huntington’s disease. Acta Neuropathol Commun. 2020 Jul 1;8(1):97. PMID: 32611447; PMCID: PMC7331280 32611447 10.1186/s40478-020-00964-z PMC7331280

[152] Rockabrand E, Slepko N, Pantalone A, et al. The first 17 amino acids of Huntingtin modulate its sub–cellular localization, aggregation and effects on calcium homeostasis. Hum Mol Genet. 2006;16(1):61–77. 17135277 10.1093/hmg/ddl440

[153] Cornett J, Cao F, Wang CE, et al. Polyglutamine expansion of huntingtin impairs its nuclear export. Nat Genet. 2005;37(2):198–204. 15654337 10.1038/ng1503

[154] Cabezas A, Pattni K, Stenmark H. Cloning and subcellular localization of a human phosphatidylinositol 3–phosphate 5–kinase, PIKfyve/Fab1. Gene. 2006;371(1):34–41. 16448788 10.1016/j.gene.2005.11.009

[155] Li X, Wang X, Zhang X, et al. Genetically encoded fluorescent probe to visualize intracellular phosphatidylinositol 3,5–bisphosphate localization and dynamics. Proc Natl Acad Sci U S A. 2013;110(52):21165–21170. 24324172 10.1073/pnas.1311864110 PMC3876232

[156] Sbrissa D, Ikonomov OC, Shisheva A. PIKfyve, a mammalian ortholog of yeast Fab1p lipid kinase, synthesizes 5–phosphoinositides. Effect of insulin. J Biol Chem. 1999;274(31):21589–21597. 10419465 10.1074/jbc.274.31.21589

[157] Ikonomov OC, Sbrissa D, Delvecchio K, et al. The phosphoinositide kinase PIKfyve is vital in early embryonic development: preimplantation lethality of PIKfyve–/– embryos but normality of PIKfyve± mice. J Biol Chem. 2011;286(15):13404–13413. 21349843 10.1074/jbc.M111.222364 PMC3075686

[158] Li X, Rydzewski N, Hider A, et al. A molecular mechanism to regulate lysosome motility for lysosome positioning and tubulation. Nat Cell Biol. 2016;18(4):404–417. 26950892 10.1038/ncb3324 PMC4871318

[159] Vines JH, Maib H, Buckley CM, et al. A PI(3,5)P2 reporter reveals PIKfyve activity and dynamics on macropinosomes and phagosomes. J Cell Biol. 2023 Sep 4;222(9):e202209077. Epub 2023 Jun 29. PMID: 37382666; PMCID: PMC10309190 37382666 10.1083/jcb.202209077 PMC10309190

[160] Yoneda A, Kanemaru K, Matsubara A, et al. Phosphatidylinositol 4,5–bisphosphate is localized in the plasma membrane outer leaflet and regulates cell adhesion and motility. Biochem Biophys Res Commun. 2020;527(4):1050–1056. 32439160 10.1016/j.bbrc.2020.05.040

[161] Tan X, Thapa N, Choi S, et al. Emerging roles of PtdIns(4,5)P2–beyond the plasma membrane. J Cell Sci. 2015;128(22):4047–4056. 26574506 10.1242/jcs.175208 PMC4712784

[162] Puri C, Renna M, Bento CF, et al. ATG16L1 meets ATG9 in recycling endosomes: additional roles for the plasma membrane and endocytosis in autophagosome biogenesis. Autophagy. 2013;10(1):182–184. 24257061 10.4161/auto.27174 PMC4389876

[163] Puri C, Renna M, Bento CF, et al. Diverse autophagosome membrane sources coalesce in recycling endosomes. Cell. 2013;154(6):1285–1299. 24034251 10.1016/j.cell.2013.08.044 PMC3791395

[164] Tan X, Thapa N, Liao Y, et al. PtdIns(4,5)P2 signaling regulates ATG14 and autophagy. Proc Natl Acad Sci U S A. 2016;113(39):10896–10901. 27621469 10.1073/pnas.1523145113 PMC5047215

[165] Baba T, Balla T. Emerging roles of phosphatidylinositol 4–phosphate and phosphatidylinositol 4,5–bisphosphate as regulators of multiple steps in autophagy. J Biochem. 2020;168(4):329–336. 32745205 10.1093/jb/mvaa089 PMC7778341

[166] Baba T, Toth DJ, Sengupta N, et al. Phosphatidylinositol 4,5–bisphosphate controls Rab7 and PLEKHM1 membrane cycling during autophagosome–lysosome fusion. EMBO J. 2019;38(8):e100312. 31368593 10.15252/embj.2018100312 PMC6463214

[167] Yanai A, Huang K, Kang R, et al. Palmitoylation of huntingtin by HIP14is essential for its trafficking and function. Nat Neurosci. 2006 Jun;9(6):1848–1858. Epub 2006 May 14. PMID: 16699508; PMCID: PMC2279235 10.1038/nn1702 PMC2279235 16699508

[168] Singaraja RR, Hadano S, Metzler M, et al. HIP14, a novel ankyrin domain–containing protein, links huntingtin to intracellular trafficking and endocytosis. Hum Mol Genet. 2002;11(23):2815–2828. 12393793 10.1093/hmg/11.23.2815

[169] Huang K, Sanders SS, Kang R, et al. Wild–type HTT modulates the enzymatic activity of the neuronal palmitoyl transferase HIP14. Hum Mol Genet. 2011 Sep 1;20(17):3356–3365. Epub 2011 Jun 2. PMID: 21636527; PMCID: PMC3153302 21636527 10.1093/hmg/ddr242 PMC3153302

[170] Butland SL, Sanders SS, Schmidt ME, et al. The palmitoyl acyltransferase HIP14 shares a high proportion of interactors with huntingtin: implications for a role in the pathogenesis of Huntington’s disease. Hum Mol Genet. 2014;23(15):4142–4160. 24705354 10.1093/hmg/ddu137 PMC4082372

[171] Stowers RS, Isacoff EY. Drosophila huntingtin–interacting protein 14 is a presynaptic protein required for photoreceptor synaptic transmission and expression of the palmitoylated proteins synaptosome–associated protein 25 and cysteine string protein. J Neurosci. 2007;27(47):12874–12883. 18032660 10.1523/JNEUROSCI.2464-07.2007 PMC6673277

[172] Sutton LM, Sanders SS, Butland SL, et al. Hip14l–deficient mice develop neuropathological and behavioural features of Huntington disease. Hum Mol Genet. 2012;22(3):452–465. 23077216 10.1093/hmg/dds441

[173] Tabata K, Imai K, Fukuda K, et al. Palmitoylation of ULK1 by ZDHHC13 plays a crucial role in autophagy. Nat Commun. 2024;15(1):7194. 39169022 10.1038/s41467-024-51402-w PMC11339336

[174] Gelman A, Rawet–Slobodkin M, Elazar Z. Huntingtin facilitates selective autophagy. Nat Cell Biol. 2015;17(3):214–215. 25720962 10.1038/ncb3125

[175] Steffan JS. Does Huntingtin play a role in selective macroautophagy? Cell Cycle. 2010;9(17):3401–3413. 20703094 10.4161/cc.9.17.12718 PMC3047613

[176] Maday S, Wallace KE, Holzbaur EL. Autophagosomes initiate distally and mature during transport toward the cell soma in primary neurons. J Cell Biol. 2012 Feb 20;196(4):407–417. Epub 2012 Feb 13. PMID: 22331844; PMCID: PMC3283992 22331844 10.1083/jcb.201106120 PMC3283992

[177] Maetzel D, Sarkar S, Wang H, et al. Genetic and chemical correction of cholesterol accumulation and impaired autophagy in hepatic and neural cells derived from niemann–pick Type C patient–specific iPS cells. Stem Cell Reports. 2014;2(6):866–880. 24936472 10.1016/j.stemcr.2014.03.014 PMC4050353

[178] Ferguson SM. Axonal transport and maturation of lysosomes. Curr Opin Neurobiol. 2018 Aug;51:45–51. doi:10.1016/j.conb.2018.02.020. Epub 2018 Mar 9. PMID: 29529416; PMCID: PMC6066426 29529416 PMC6066426

[179] Caviston JP, Ross JL, Antony SM, et al. Huntingtin facilitates dynein/dynactin–mediated vesicle transport. Proc Natl Acad Sci U S A. 2007;104(24):10045–10050. 17548833 10.1073/pnas.0610628104 PMC1891230

[180] Li S–H, Gutekunst C–A, Hersch SM, et al. Interaction of huntingtin–associated protein with dynactin P150 glued. J Neurosci. 1998 Feb 15;18(4):1261–1269. PMID: 9454836; PMCID: PMC6792727 9454836 10.1523/JNEUROSCI.18-04-01261.1998 PMC6792727

[181] Cason SE, Carman PJ, Van Duyne C, et al. Sequential dynein effectors regulate axonal autophagosome motility in a maturation–dependent pathway. J Cell Biol. 2021;220(7). doi:10.1083/jcb.202010179 PMC8142281 34014261

[182] Özkan N, Koppers M, van Soest I, et al. ER – lysosome contacts at a pre–axonal region regulate axonal lysosome availability. Nat Commun. 2021 Jul 23;12(1):4493. PMID: 34301956; PMCID: PMC8302662 34301956 10.1038/s41467-021-24713-5 PMC8302662

[183] Tammineni P, Ye X, Feng T, et al., Impaired retrograde transport of axonal autophagosomes contributes to autophagic stress in Alzheimer’s disease neurons. Elife. 2017 Jan 13; 6:e21776. PMID: 28085665; PMCID: PMC5235353 28085665 10.7554/eLife.21776 PMC5235353

[184] Holzer E, Martens S, Tulli S. The role of ATG9 vesicles in autophagosome biogenesis. J Mol Biol. 2024 Aug 1;436(15):168489. Epub 2024 Feb 10. Erratum in: J Mol Biol. 2024 Dec 1;436(23):168849. doi: 10.1016/j.jmb.2024.168849. PMID: 38342428 38342428

[185] Olivas TJ, Wu Y, Yu S, et al. ATG9 vesicles comprise the seed membrane of mammalian autophagosomes. J Cell Biol. 2023 Jul 3;222(7):e202208088. Epub 2023 Apr 28. PMID: 37115958; PMCID: PMC10148236 37115958 10.1083/jcb.202208088 PMC10148236

[186] Binotti B, Ninov M, Cepeda AP, et al. ATG9 resides on a unique population of small vesicles in presynaptic nerve terminals. Autophagy. 2023;20(4):883–901. 37881948 10.1080/15548627.2023.2274204 PMC11062364

[187] Yang S, Park D, Manning L, et al. Presynaptic autophagy is coupled to the synaptic vesicle cycle via ATG–9. Neuron. 2022;110(5):824–840.e10. 35065714 10.1016/j.neuron.2021.12.031 PMC9017068

[188] Legakis JE, Yen WL, Klionsky DJ. A cycling protein complex required for selective autophagy. Autophagy. 2007;3(5):422–432. 17426440 10.4161/auto.4129

[189] Kannangara AR, Poole DM, McEwan CM, et al. BioID reveals an ATG9A interaction with ATG13–ATG101 in the degradation of p62/SQSTM1–ubiquitin clusters. EMBO Rep. 2021;22(10):e51136. 34369648 10.15252/embr.202051136 PMC8490997

[190] Ren X, Nguyen TN, Lam WK, et al. Structural basis for ATG9A recruitment to the ULK1 complex in mitophagy initiation. Sci Adv. 2023;9(7):eadg2997. 36791199 10.1126/sciadv.adg2997 PMC9931213

[191] Popovic D, Dikic I. TBC1D5 and the AP2 complex regulate ATG9 trafficking and initiation of autophagy. EMBO Rep. 2014;15(4):392–401. 24603492 10.1002/embr.201337995 PMC3989670

[192] Boucrot E, Saffarian S, Zhang R, et al. Roles of AP–2 in clathrin–mediated endocytosis. PLoS One. 2010;5(5):e10597. 20485680 10.1371/journal.pone.0010597 PMC2868873

[193] Martin TFJ. Role of PI(4,5)P(2) in vesicle exocytosis and membrane fusion. Subcell Biochem. 2012;59:111–130. 22374089 10.1007/978-94-007-3015-1_4 PMC3978774

[194] Metzler M, Legendre–Guillemin V, Gan L, et al. HIP1 functions in clathrin–mediated endocytosis through binding to clathrin and adaptor protein 2. J Biol Chem. 2001;276(42):39271–39276. 11517213 10.1074/jbc.C100401200

[195] Imoto Y, Raychaudhuri S, Ma Y, et al. Dynamin is primed at endocytic sites for ultrafast endocytosis. Neuron. 2022;110(17):2815–2835.e13. 35809574 10.1016/j.neuron.2022.06.010 PMC9464723

[196] El–Daher MT, Hangen E, Bruyère J, et al. Huntingtin proteolysis releases non–polyQ fragments that cause toxicity through dynamin 1 dysregulation. EMBO J. 2015;34(17):2255–2271. 26165689 10.15252/embj.201490808 PMC4585462

[197] Kalchman MA, Koide HB, McCutcheon K, et al. HIP1, a human homologue of S. cerevisiae Sla2p, interacts with membrane–associated huntingtin in the brain. Nat Genet. 1997;16(1):44–53. 9140394 10.1038/ng0597-44

[198] Mishra SK, Agostinelli NR, Brett TJ, et al. Clathrin– and AP–2–binding sites in HIP1 uncover a general assembly role for endocytic accessory proteins. J Biol Chem. 2001;276(49):46230–46236. 11577110 10.1074/jbc.M108177200

[199] Dent EW, Gupton SL, Gertler FB. The growth cone cytoskeleton in axon outgrowth and guidance. Cold Spring Harb Perspect Biol. 2011 Mar 1;3(3):a001800. PMID: 21106647; PMCID: PMC3039926 21106647 10.1101/cshperspect.a001800 PMC3039926

[200] Kole AJ, Annis RP, Deshmukh M. Mature neurons: equipped for survival. Cell Death Dis. 2013;4(6):e689. 23807218 10.1038/cddis.2013.220 PMC3702294

[201] Geoffroy CG, Hilton BJ, Tetzlaff W, et al. Evidence for an age–dependent decline in axon regeneration in the adult mammalian central nervous system. Cell Rep. 2016;15(2):238–246. 27050519 10.1016/j.celrep.2016.03.028 PMC5050004

[202] Sutherland TC, Sefiani A, Horvat D, et al. Age–dependent decline in neuron growth potential and mitochondria functions in cortical neurons. Cells. 2021;10(7):1625. 34209640 10.3390/cells10071625 PMC8306398

[203] van Erp S, van Berkel AA, Feenstra EM, et al. Age–related loss of axonal regeneration is reflected by the level of local translation. Exp Neurol. 2021;339:113594. 33450233 10.1016/j.expneurol.2020.113594 PMC8024785

[204] Aversano S, Caiazza C, Caiazzo M. Induced pluripotent stem cell–derived and directly reprogrammed neurons to study neurodegenerative diseases: the impact of aging signatures. Front Aging Neurosci. 2022;14:1069482. 36620769 10.3389/fnagi.2022.1069482 PMC9810544

[205] Mertens J, Reid D, Lau S, et al. Aging in a dish: iPSC–derived and directly induced neurons for studying brain aging and age–related Neurodegenerative diseases. Annu Rev Genet. 2018;52(1):271–293. 30208291 10.1146/annurev-genet-120417-031534 PMC6415910

[206] Inagaki E, Yoshimatsu S, Okano H. Accelerated neuronal aging in vitro ∼melting watch ∼. Front Aging Neurosci. 2022;14:868770. 36016855 10.3389/fnagi.2022.868770 PMC9397486

[207] Mertens J, Paquola ACM, Ku M, et al. Directly reprogrammed human neurons retain aging–associated transcriptomic signatures and reveal age–related nucleocytoplasmic defects. Cell Stem Cell. 2015;17(6):705–718. 26456686 10.1016/j.stem.2015.09.001 PMC5929130

[208] Wang G, Liu X, Gaertig MA, et al. Ablation of huntingtin in adult neurons is nondeleterious but its depletion in young mice causes acute pancreatitis. Proc Natl Acad Sci U S A. 2016;113(12):3359–3364. 26951659 10.1073/pnas.1524575113 PMC4812735

[209] Van Raamsdonk JM, Pearson J, Rogers DA, et al. Loss of wild–type huntingtin influences motor dysfunction and survival in the YAC128 mouse model of Huntington disease. Hum Mol Genet. 2005 May 15;14(10):1379–1392. Epub 2005 Apr 13. PMID: 15829505 15829505 10.1093/hmg/ddi147

[210] Ban BK, Jun MH, Ryu HH, et al. Autophagy negatively regulates early axon growth in cortical neurons. Mol Cell Biol. 2013 Oct;33(19):3907–3919. Epub 2013 Aug 5. PMID: 23918799; PMCID: PMC3811863 23918799 10.1128/MCB.00627-13 PMC3811863

[211] Li H, Li SH, Yu ZX, et al. Huntingtin aggregate–associated axonal degeneration is an early pathological event in Huntington’s disease mice. J Neurosci. 2001;21(21):8473–8481. 11606636 10.1523/JNEUROSCI.21-21-08473.2001 PMC6762783

[212] Pryor WM, Biagioli M, Shahani N, et al. Huntingtin promotes mTORC1 signaling in the pathogenesis of Huntington’s disease. Sci Signal. 2014;7(349):ra103. 25351248 10.1126/scisignal.2005633

[213] Ravikumar B, Vacher C, Berger Z, et al. Inhibition of mTOR induces autophagy and reduces toxicity of polyglutamine expansions in fly and mouse models of Huntington disease. Nat Genet. 2004;36(6):585–595. 15146184 10.1038/ng1362

[214] Ma TC, Buescher JL, Oatis B, et al. Metformin therapy in a transgenic mouse model of Huntington’s disease. Neurosci Lett. 2006;411(2):98–103. 17110029 10.1016/j.neulet.2006.10.039

[215] Ju TC, Chen HM, Lin JT, et al. Nuclear translocation of AMPK–alpha1 potentiates striatal neurodegeneration in Huntington’s disease. J Cell Biol. 2011;194(2):209–227. 21768291 10.1083/jcb.201105010 PMC3144412

[216] Chou SY, Lee YC, Chen HM, et al. CGS21680 attenuates symptoms of Huntington’s disease in a transgenic mouse model. J Neurochem. 2005;93(2):310–320. 15816854 10.1111/j.1471-4159.2005.03029.x

[217] Wold MS, Lim J, Lachance V, et al. ULK1–mediated phosphorylation of ATG14 promotes autophagy and is impaired in Huntington’s disease models. Mol Neurodegener. 2016;11(1):76. 27938392 10.1186/s13024-016-0141-0 PMC5148922

[218] Lim J, Lachenmayer ML, Wu S, et al. Proteotoxic stress induces phosphorylation of p62/SQSTM1 by ULK1 to regulate selective autophagic clearance of protein aggregates. PLoS Genet. 2015;11(2):e1004987. 25723488 10.1371/journal.pgen.1004987 PMC4344198

[219] Mealer RG, Murray AJ, Shahani N, et al. Rhes, a striatal–selective protein implicated in Huntington disease, binds beclin–1 and activates autophagy. J Biol Chem. 2013;289(6):3547–3554. 24324270 10.1074/jbc.M113.536912 PMC3916556

[220] Walter C, Clemens LE, Müller AJ, et al. Activation of AMPK–induced autophagy ameliorates Huntington disease pathology in vitro. Neuropharmacology. 2016;108:24–38. 27133377 10.1016/j.neuropharm.2016.04.041

[221] Yang J, Chen X, Xu H. SQSTM1/p62 droplet –mediated autophagosome formation:insights into Huntington disease. Autophagy. 2021;17(10):3256–3259. 34281469 10.1080/15548627.2021.1953820 PMC8525954

[222] Ji CH, Kim HY, Lee MJ, et al. The AUTOTAC chemical biology platform for targeted protein degradation via the autophagy–lysosome system. Nat Commun. 2022;13(1):904. 35173167 10.1038/s41467-022-28520-4 PMC8850458

[223] Prowse ENP, Turkalj BA, Sébastien M, et al. Huntingtin polyglutamine expansions misdirect axonal transport by perturbing motor and adaptor recruitment. bioRxiv 2024.04.12.589210; d oi.o rg/1 0.1101/2024.04.12.589210 10.1016/j.isci.2026.115748 PMC13194641 42181265

[224] Li XJ, Li SH, Sharp AH, et al. A huntingtin–associated protein enriched in brain with implications for pathology. Nature. 1995;378(6555):398–402. 7477378 10.1038/378398a0

[225] Shimojo M. Huntingtin regulates RE1–silencing transcription factor/neuron–restrictive silencer factor (REST/NRSF) nuclear trafficking indirectly through a complex with REST/NRSF–interacting LIM domain protein (RILP) and dynactin p150 glued. J Biol Chem. 2008;283(50):34880–34886. 18922795 10.1074/jbc.M804183200 PMC2596380

[226] Del Toro D, Alberch J, Lázaro–Diéguez F, et al. Mutant huntingtin impairs post–Golgi trafficking to lysosomes by delocalizing optineurin/Rab8 complex from the Golgi apparatus. Mol Biol Cell. 2009;20(5):1478–1492. 19144827 10.1091/mbc.E08-07-0726 PMC2649260

[227] Jeong SJ, Stitham J, Evans TD, et al. Trehalose causes low–grade lysosomal stress to activate TFEB and the autophagy–lysosome biogenesis response. Autophagy. 2021;17(11):3740–3752. 33706671 10.1080/15548627.2021.1896906 PMC8632292

[228] Tanaka M, Machida Y, Niu S, et al. Trehalose alleviates polyglutamine–mediated pathology in a mouse model of Huntington disease. Nat Med. 2004;10(2):148–154. 14730359 10.1038/nm985

[229] Baltazar GC, Guha S, Lu W, et al. Acidic nanoparticles are trafficked to lysosomes and restore an acidic lysosomal pH and degradative function to compromised ARPE–19 cells. PLoS One. 2012;7(12):e49635. 23272048 10.1371/journal.pone.0049635 PMC3525582

[230] Lo CH, O’Connor LM, Loi GWZ, et al. Acidic nanoparticles restore lysosomal acidification and rescue metabolic dysfunction in pancreatic β–cells under lipotoxic conditions. ACS Nano. 2024 Jun 18;18(24):15452–15467. Epub 2024 Jun 3. PMID: 38830624; PMCID: PMC11192035 38830624 10.1021/acsnano.3c09206 PMC11192035

[231] Arotcarena ML, Soria FN, Cunha A, et al. Acidic nanoparticles protect against α–synuclein–induced neurodegeneration through the restoration of lysosomal function. Aging Cell. 2022;21(4):e13584. 35318803 10.1111/acel.13584 PMC9009122

[232] Ananbeh H, Vodicka P, Kupcova Skalnikova H. Emerging roles of exosomes in Huntington’s Disease. Int J Mol Sci. 2021;22(8):4085. 33920936 10.3390/ijms22084085 PMC8071291

[233] Xing H, Tan J, Miao Y, et al. Crosstalk between exosomes and autophagy: a review of molecular mechanisms and therapies. J Cell Mol Med. 2021;25(5):2297–2308. 33506641 10.1111/jcmm.16276 PMC7933923

[234] Ananbeh H, Novak J, Juhas S, et al. Huntingtin co–isolates with small extracellular vesicles from blood plasma of TgHD and KI–HD pig models of Huntington’s disease and human blood plasma. Int J Mol Sci. 2022;23(10):5598. 35628406 10.3390/ijms23105598 PMC9147436

[235] Jeon I, Cicchetti F, Cisbani G, et al. Human–to–mouse prion–like propagation of mutant huntingtin protein. Acta Neuropathol. 2016;132(4):577–592. 27221146 10.1007/s00401-016-1582-9 PMC5023734

